# Multiscale modeling of influenza A virus replication in cell cultures predicts infection dynamics for highly different infection conditions

**DOI:** 10.1371/journal.pcbi.1006819

**Published:** 2019-02-19

**Authors:** Daniel Rüdiger, Sascha Young Kupke, Tanja Laske, Pawel Zmora, Udo Reichl

**Affiliations:** 1 Max Planck Institute for Dynamics of Complex Technical Systems, Magdeburg, Germany; 2 Chair of Bioprocess Engineering, Institute of Process Engineering, Faculty of Process & Systems Engineering, Otto-von-Guericke University, Magdeburg, Germany; University of Texas at Austin, UNITED STATES

## Abstract

Influenza A viruses (IAV) are commonly used to infect animal cell cultures for research purposes and vaccine production. Their replication is influenced strongly by the multiplicity of infection (MOI), which ranges over several orders of magnitude depending on the respective application. So far, mathematical models of IAV replication have paid little attention to the impact of the MOI on infection dynamics and virus yields. To address this issue, we extended an existing model of IAV replication in adherent MDCK cells with kinetics that explicitly consider the time point of cell infection. This modification does not only enable the fitting of high MOI measurements, but also the successful prediction of viral release dynamics of low MOI experiments using the same set of parameters. Furthermore, this model allows the investigation of defective interfering particle (DIP) propagation in different MOI regimes. The key difference between high and low MOI conditions is the percentage of infectious virions among the total virus particle release. Simulation studies show that DIP interference at a high MOI is determined exclusively by the DIP content of the seed virus while, in low MOI conditions, it is predominantly controlled by the *de novo* generation of DIPs. Overall, the extended model provides an ideal framework for the prediction and optimization of cell culture-derived IAV manufacturing and the production of DIPs for therapeutic use.

## Author summary

Influenza is a contagious respiratory disease that severely affects several million people each year. Vaccination can provide protection against the infection, but vaccine composition has to be adjusted regularly to remain effective against this fast evolving pathogen. While influenza vaccines are mostly produced in embryonated chicken eggs, cell culture-based vaccine production is developing as an alternative providing controlled process conditions in closed systems, better scalability, and a short response time in case of pandemic outbreaks. Here, we employ a computational model to describe underlying mechanisms during the IAV infection in adherent MDCK cells. Special attention was paid on the influence of the MOI on virus spread in cell populations. Although dynamics between infections with high and low amounts of infecting virions differ significantly, our model closely captures both scenarios. Furthermore, our results provide insights into IAV-induced apoptosis and the switch from transcription to replication in intracellular IAV replication. Additionally, model simulations indicate how virus particle release is regulated, and what impact defective interfering particles have on virus replication in different infection conditions. Taken together, we developed a computational model that enables detailed analyses of IAV replication dynamics in animal cell culture.

## Introduction

Influenza A virus (IAV) is an enveloped, segmented, single-stranded RNA virus that infects humans, livestock and various wild animals. IAV has been in the focus of basic and applied research for decades, but still poses a considerable risk to public health. Current annual epidemics cause up to five million severe infections and at least half a million deaths worldwide [1]. Historically, influenza pandemics have the potential for hazardous impacts with up to one hundred million deaths [2]. Vaccination provides protection against infection but vaccine composition has to be adapted seasonally to the most prevalent strains.

Influenza vaccine is manufactured mainly in embryonated chicken eggs, an established process dating back to the middle of the 20th century. The egg-based vaccine production is constrained by scale-up restrictions, low yields for some virus strains, and potential allergic reactions [3–5]. Cell culture-based production is considered as an alternative to overcome these limitations. Cell cultures provide scalability and controlled sterile process settings in bioreactors [3,4]. However, cell culture-based influenza vaccine production is still facing challenges regarding yields, process costs and the adaptation of seed viruses to the desired cell line. Deeper insights into the virus replication and spread in cell cultures in different infection conditions are vital to overcome these challenges.

In general, infection spread in cell cultures is related to the number of infectious virus particles per cell (multiplicity of infection, MOI) while process yields are directly correlated to the cell concentration and the number of virions released by a cell (cell-specific virus yield). Cells infected by IAV release a mixture of different particles, including (I) infectious, fully functional particles, (II) non-infectious particles with various defects prohibiting virus entry and reproduction, (III) non-infectious defective interfering particles (DIPs) that may impede viral infection and reduce virus yields [6–9], and (IV) deformed, broken or empty virions [4]. Understanding the interplay between these particles and how they impact seed virus quality, infection conditions, and virus yields could be a key factor for the optimization of cell culture-based influenza vaccine production.

Mathematical modeling has proven to be a valuable tool for the investigation and analysis of biological systems. IAV infection dynamics was examined in depth with model-based studies that predominantly focus on the cell population level disregarding processes inside an infected cell [10–13]. Such models mostly investigate the host cell immune response and the prevention of virus spreading, which limits their applicability for cell culture-based influenza vaccine production. Other studies investigated intracellular processes, i.e. virus entry and replication, with deterministic [14,15] and stochastic approaches [16,17]. However, by disregarding virus spread these models are not able to describe infection dynamics in a cell culture. Recently, mathematical models were employed to examine the effect of DIPs on IAV infection [18–20]. Furthermore, the periodic accumulation of DIPs was observed in a continuous cell culture-based virus production setup [3]. However, mathematical models of IAV replication have paid little attention to process and infection conditions, e.g. the MOI.

Differences in the MOI can exert a significant influence on the infection dynamics of a single cell and the whole cell population. Furthermore, the MOI is an important factor for yields in animal cell cultures as shown in several experimental studies [21–23]. Testing different infection conditions for IAV production, high MOI regimes lead to a fast drop of viable cell concentrations and lower virus yields than infections in low MOI conditions. However, the optimal MOI for virus production is strain-dependent [22] and presumably affected by various process parameters and the cell lines used. Accordingly, a mathematical model that accounts for the influence of different MOIs on intra- and extracellular processes during IAV infection in cell cultures could certainly support the optimization of vaccine production processes.

In our group a multiscale model describing IAV infection of mammalian cell cultures was developed recently [24]. Here, we present an extended version of this model that particularly considers effects of infection conditions on virus dynamics. To this end, we introduce new mechanisms that adjust virus-induced apoptosis, virus release, and inhibition of viral mRNA synthesis to describe infection dynamics in different MOI regimes. We demonstrate that the extended model closely describes previously published viral RNA, virus release, and cell population measurements from adherent MDCK cells infected at a high MOI [4]. We then use the model to successfully predict virus release dynamics for cell culture infections performed in lower MOI conditions. Finally, we show how the MOI in a cell culture evolves during the infection and how this influences DIP propagation.

## Results

### Multiscale model of influenza virus infection

The model presented in this publication is based on a multiscale model of IAV infection in animal cell cultures by Heldt et al. [24]. This original model combines intracellular virus replication with virus propagation on the cell population level. On the intracellular level, various steps of virus replication including viral RNA synthesis, viral protein translation, and virion release are implemented in detail. The extracellular level describes population kinetics of uninfected cells, infected cells, apoptotic cells, and free virions (infectious virus particles). The two levels of viral infection are linked by connecting the intracellular dynamics to a segregated population of infected cells. This segregation considers the time that has passed since cells were infected, i.e. the infection age *τ*. Virion release on the intracellular level, which is dependent on the infection age, is linked to the segregated infected cell population. Thus, the intra- and extracellular level of IAV infection can be simulated simultaneously.

**Fig 1:**
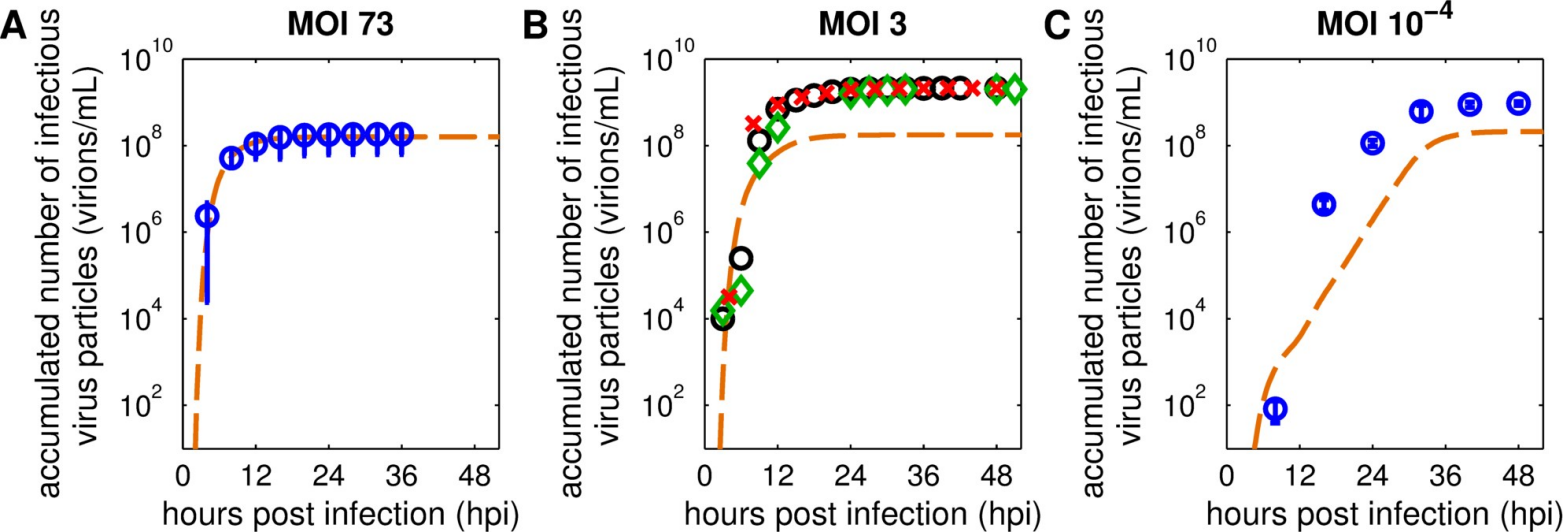
The original model fails to capture virus particle release dynamics for different infection conditions. Curves depict model simulations of infectious virus particles released during MDCK cell infections with influenza A/PR/8/34 (H1N1) generated by the original multiscale model of Heldt et al. [24]. Results for MOI 73 were adapted from [4], experiments for MOI 3 and 10^−4^ were conducted following the same protocol. Infections at MOI 73 and MOI 3 were performed with a PR8 strain from the National Institute for Biological Standards and Control (NIBSC); for the infection at MOI 10^−4^ a PR8 strain from the Robert Koch Institute (RKI) was used. Symbols represent the accumulated number of infectious virus particles quantified by the TCID_50_ assay. (A+C) Error bars indicate standard deviations of three independent experiments, time courses of three individual experiments in (B) are shown separately (○, ◇, □). Model parameters were fitted to experimental results obtained with (A) an MOI of 73 [4]. Relevant initial conditions in (B+C) were adjusted to the respective infection conditions.

The original model was calibrated to measurements from two separate experiments performed at a high MOI for the intracellular virus dynamics and a low MOI for the extracellular part. Fortunately, in a recent publication, both intra- and extracellular IAV replication dynamics in MDCK cells were analyzed in high MOI conditions [4]. To that end, an MOI of 10 PFU (plaque forming units) per cell was used, which correlated to an MOI of about 73 based on TCID_50_ assay results. This high virus load was applied to achieve a single-cycle infection and resulted in a rapid infection of cells. Consequently, all cells were infected in a confined time frame leading to similar cell infection ages. This enabled a profound investigation of the cell population dynamics over the time course of infection as we do not observe a mixture of cells at different stages of infection. A recalibration of the original model to measurements provided by this newer study showed mixed results. While levels of viral cRNA, vRNA (S1 Fig) and virion release (Fig 1A) could be reproduced, cell population and viral mRNA dynamics (S1 Fig) differed considerably from the experimental data. Additionally, model predictions performed with the original model fitted for high MOI measurements from [4] did not capture viral release dynamics observed in new infection experiments performed at lower MOIs (Fig 1B and 1C). We therefore decided to augment the original model to enable the description of IAV infection dynamics for a wider range of MOI conditions, e.g. in low MOI regimes used in cell culture-based influenza vaccine production.

### Model extension

**Fig 2:**
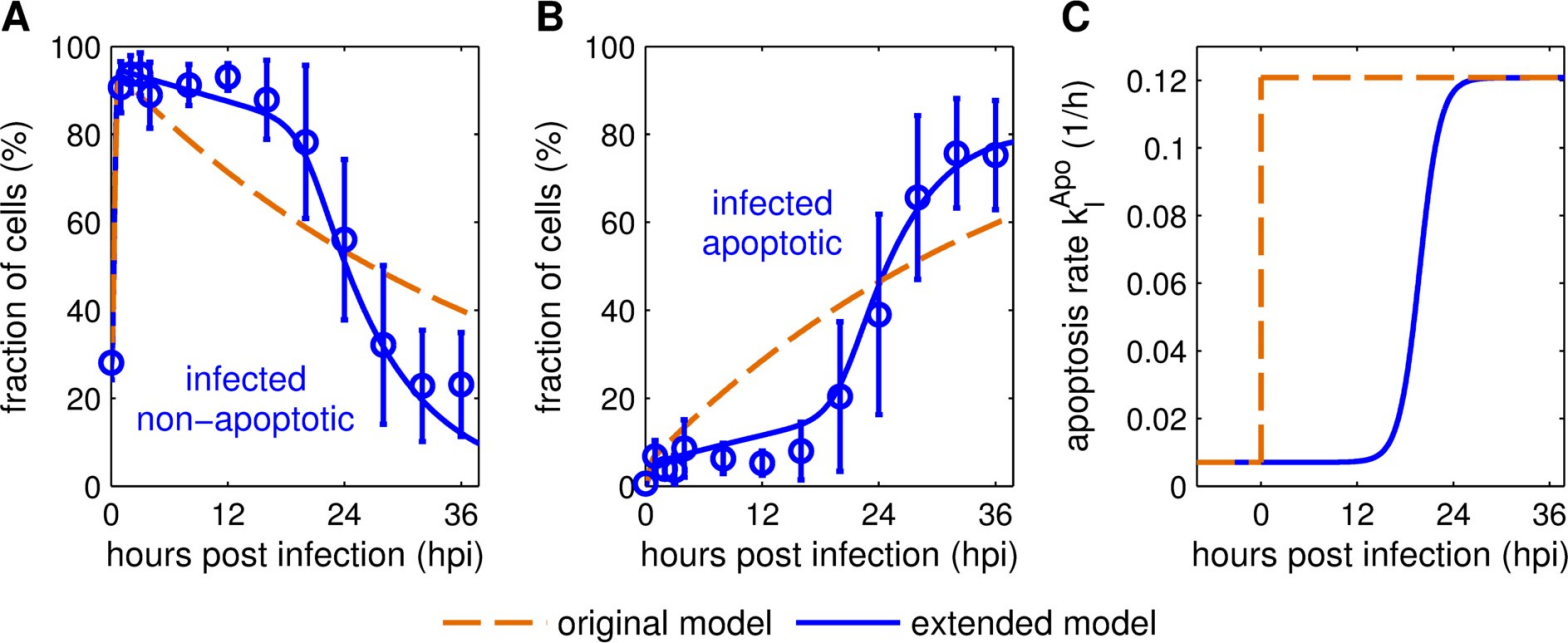
Logistic apoptosis rate enables reproduction of cell population dynamics. Model fits to cell population measurements from MDCK cell cultures infected with influenza A/PR/8/34 (H1N1) at an MOI of 73 [4]. Mean percentage values of (A) infected, non-apoptotic and (B) infected, apoptotic cells from three independent experiments are shown. Curves represent simulations of the original [24] (dashed) and the extended (solid) multiscale model. (C) Comparison of infected, non-apoptotic cell apoptosis rate dynamics using a stepwise (original model, [24]) and logistically (extended model) increasing virus-induced apoptosis rate $k_{I}^{\mathrm{Apo}}$. Hours post infection in (A) and (B) refer to the time passed since the cell culture was infected while in (C) it corresponds to the individual infection time point of a cell.

First, we focused on the description of cell population dynamics during the IAV infection of cell cultures. On the extracellular level the model considers uninfected cells, infected cells and apoptotic cells of both cell populations. Previous measurements of a single-cycle IAV infection of adherent MDCK cells showed that apoptotic cells start to accumulate about 16 hours post infection (hpi) [4]. To describe this delay, which is not considered in the original model (Fig 2A and 2B), we adjusted the apoptosis rate of infected cells (Eq (9)). The original model assumed an exponential distribution of the survival time of infected cells, which was implemented by a stepwise increase of the apoptosis rate to a fixed value after infection [24]. In a previous study, Holder et al. suggested that normal distributions are well suited to describe the time cells spend in a specific state [25]. However, to introduce an infection age-dependent apoptosis rate, the cumulative density function of the normal distribution is required. This cumulative density function utilizes the Gauss error function and does not have a closed analytical form of estimation [26]. Therefore, we checked other approaches that approximate normal distribution-like dynamics while still being easy to handle, i.e. a logistic function, Hill kinetics, and a Gompertz function, based on their capability to introduce sigmoid dynamics. All of these functions described the time course of virus-induced apoptosis relatively well (S3 Fig).

Finally, we decided to use a logistic function to describe the apoptosis rate of infected cells (Eq (9)) as it provides two benefits. The logistic function allowed the best fit of the experimental data and, in contrast to a Hill kinetic, the introduced parameters can be readily interpreted, i.e. *τ*_Apo_ as the time after cell infection at which the rate of virus-induced apoptosis reaches its half-maximum, and *ν*_Apo_ as a factor that describes the time required from cell infection until the full activation of the apoptosis mechanism. Fig 2C shows the difference between both scenarios. The original model uses an apoptosis rate that instantly increases after cell infection while the extended model establishes a delayed, gradually increasing rate. Hence, the implementation of an infection age-dependent rate of virus-induced apoptosis enables a better description of cell population dynamics observed in a single-cycle IAV infection of adherent MDCK cells (Fig 2A and 2B) [4].

**Fig 3:**
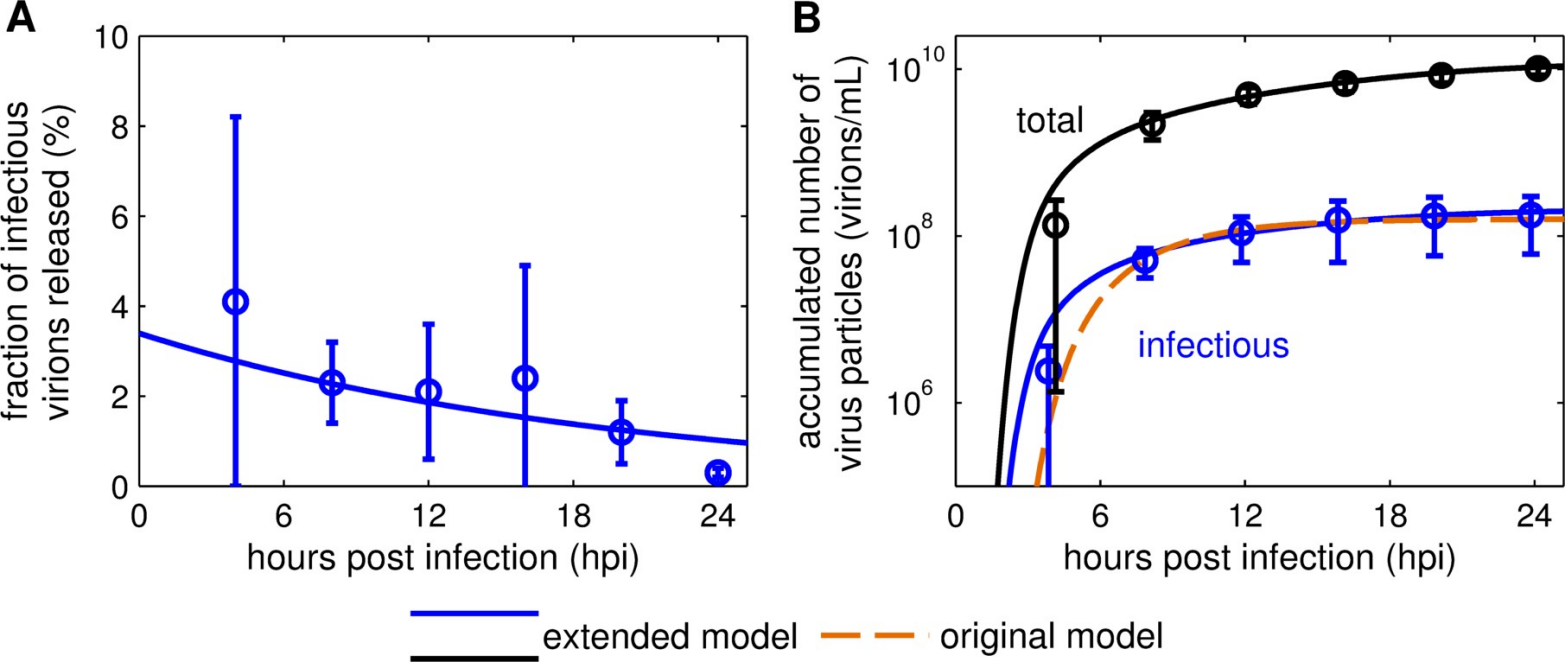
Relation of infectious to total virus particles released. (A) The fraction of infectious virions released, (B) the accumulated number of infectious and total virus particles were taken from three individual experiments performed in [4]. In brief, adherent MDCK cells were infected with influenza A/PR/8/34 (H1N1) at an MOI of 73. Cumulative viral titers were determined by TCID_50_ assay and HA assay, respectively. The solid lines represent the model fit to the data with the extended model, (B) the dashed line depicts the model fit with the original model [24].

Next, we extended the original model to consider the number of infectious virions (determined by infectivity assays, e.g. TCID_50_) and the total number of virus particles (determined by the HA assay) separately. This characteristic was not accounted for in the original model, which solely focused on “virions” (infectious virus particles). However, the concentration of total virus particles holds valuable information for influenza virus production as it correlates with process yields for manufacturing of inactivated vaccines. Accordingly, we implemented the virus particle release of an individual infected cell (Eq (2)) and defined the release of infectious virions as a fraction of the overall release (Eq (3)). As shown in [4], measurements of virions released indicate that the ratio of infectious to total virus particles release is not constant over the course of infection. In MDCK cell cultures infected with A/Puerto Rico/8/34 (PR8) at an MOI of 73, the initial fraction of infectious virions released (FIVR) was about 4% and decreased over time to about 0.3% (Fig 3A). As a result, most virions released during late infection were non-infectious. The variable *F*_Par_(*τ*) (Eq (4)) describes the FIVR by an infected cell at a certain infected cell age *τ*. Generally, the FIVR represents influencing factors that affect the quality of the released virus particles. Plausible effects impacting it are the accumulation of DIPs or limited precursors for viral protein and RNA synthesis due to a rapid replication of the virus. The dynamics of *F*_Par_ is defined as a first order degradation to reproduce the observation that infected cells release higher percentages of infectious virus particles immediately after infection compared to later time points (Fig 3A).

Using this infection age-dependent variable, the model captures both infectious and overall virus particles released by an individual infected cell (Fig 3B). To describe the total amount of virus particles on the population level, we introduced the variable $P_{\mathrm{tot}}^{\mathrm{Rel}}$ (Eq (6)), which can be correlated to IAV production yields. Thus, these model extensions enable the description of both the cell-specific yield and overall viral titers during IAV production in cell cultures.

**Fig 4:**
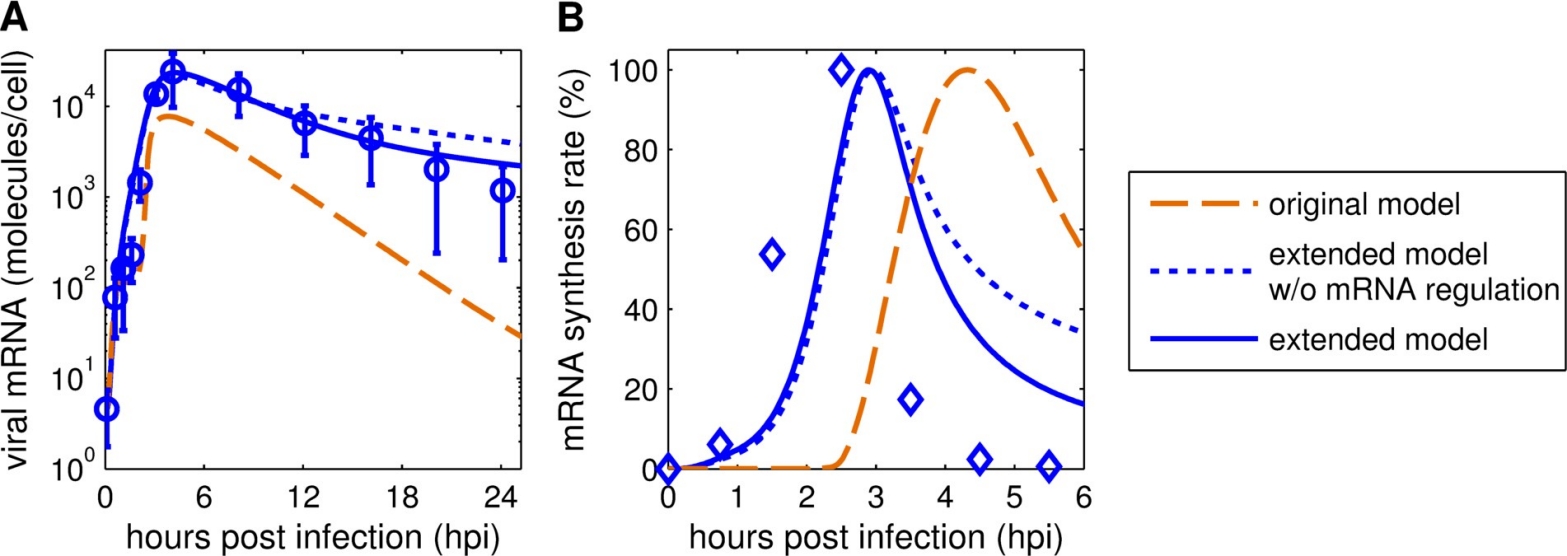
Implementation of an inhibition of viral mRNA synthesis improves description of viral mRNA dynamics. (A) Model fits to viral mRNA measurements of MDCK cultures infected with influenza A/PR/8/34 (H1N1) at an MOI of 73 based on TCID_50_ [4]. Error bars indicate standard deviations of three individual experiments. (B) Percentage of viral mRNA synthesis rate for different model versions, experimental data were adapted from [27]. In short, BHK-21 cells were infected by influenza virus (WSN strain) at 10 to 20 PFU per cell.

The final step of model extension was to take details of intracellular viral mRNA dynamics into account. After implementation of the aforementioned changes, the description of viral mRNA dynamics still showed deviations from the measurements (Fig 4A, dotted line). While the initial accumulation of viral mRNA and the resulting peak could be captured, its following degradation could not be fully reproduced. Previous experimental studies of IAV infection in high MOI conditions indicated that a complete shutdown of viral mRNA synthesis occurs at about 6 hpi [27]. The original model did not describe such a rapid shutdown and viral mRNA synthesis continued during late infection (Fig 4B). To capture such dynamics another extension of the mathematical model, i.e. the inhibition of viral mRNA synthesis, was necessary.

Viral mRNA is transcribed from viral genome templates by hijacking the mRNA transcription mechanism of the host cell. To that end, viral RNA-dependent RNA polymerase (RdRp) binds to the cellular polymerase 2 (Pol II) and snatches precursors of cellular mRNA, which are then used to transcribe viral mRNA [28]. Recent studies have shown that RdRp has two different mechanisms of interaction with Pol II [29,30]. When RdRp carries a copy of the viral genome, the binding to Pol II induces viral mRNA synthesis. However, binding of Pol II without this template leads to the specific degradation of Pol II. The reduction of Pol II levels impedes viral mRNA transcription and could lead to a shutdown as observed in [27]. Additionally, simulation results of RdRp accumulation coincide with the decrease of viral mRNA synthesis, further supporting this hypothesis.

To keep kinetics simple, we implemented an inhibition of viral mRNA synthesis by free RdRp (Eq (5)). This indirect approach was used as the inclusion of a variable for Pol II without supporting measurements for parameter estimation would have unnecessarily increased model complexity. As before, accounting for this mechanism in the extended model improved the fit to measurements for an MOI of 73, which is affirmed by a lower sum of squared residuals and the Akaike information criterion [31] (Table 1). Furthermore, the extended model with inhibition of the viral mRNA synthesis allowed a more precise description of viral mRNA levels at time points later than 12 hpi (Fig 4A) and the simulated mRNA synthesis rate showed dynamics similar to previous studies [27] (Fig 4B).

**Table 1:** Evaluation of the model fits performed for the extended model with and without inhibition of viral mRNA synthesis.

	extended model w/o inhibition of viral mRNA synthesis	extended model
SSR	1.96	1.88
AIC	-437.2	-440.4

SSR: sum of squared residuals (error of each variable normalized to the respective maximum measurement value); AIC: Akaike information criterion

### Model calibration for high MOI measurements

The extended model containing the adjusted apoptosis dynamics, virus particle release, and inhibition of viral mRNA synthesis was calibrated to previously published measurements from IAV infections of adherent MDCK cells performed at an MOI of 73 [4]. Experimental data of the intracellular level (i.e. viral RNA dynamics) and the extracellular level (i.e. cell population and viral release dynamics) were utilized. The model was fit to both data sets simultaneously.

Model simulation is in good agreement with the experimental data on both the intra- and the extracellular level (S1 Fig). In particular, the early accumulation of viral mRNA and cRNA can be captured closely. However, the extended model underestimates the levels of vRNA between 3 to 8 hpi (S1 Fig). The cell population dynamics, i.e. the fast progress of cell infection and initiation of virus-induced apoptosis, are described well. All cells are infected at 1 hpi due to the high MOI conditions and infected cells start to undergo apoptosis around 16 hpi. The model slightly overestimates the onset of viral release on the population level, but reproduces later measurements for infectious virus particles and the total number of virions. The delay between cell infection and first virus release at MOI 73 constitutes roughly 3 h (Fig 3B), indicating that the high viral load results in fast uptake and intracellular replication of virions.

### Model prediction of low MOI dynamics

In a next step, we investigated the predictive power of the extended model calibrated for MOI 73 measurements [4], by challenging it with new experimental data from MDCK cell infections performed in lower MOI conditions. To that end, we conducted IAV infections at MOIs of 3 and 10^−4^ based on TCID_50_ to cover a broad range of infection scenarios. We performed the experiments according to the protocol used for the high MOI infections of MDCK cells [4] and measured time courses of infectious and total virus release.

**Fig 5:**
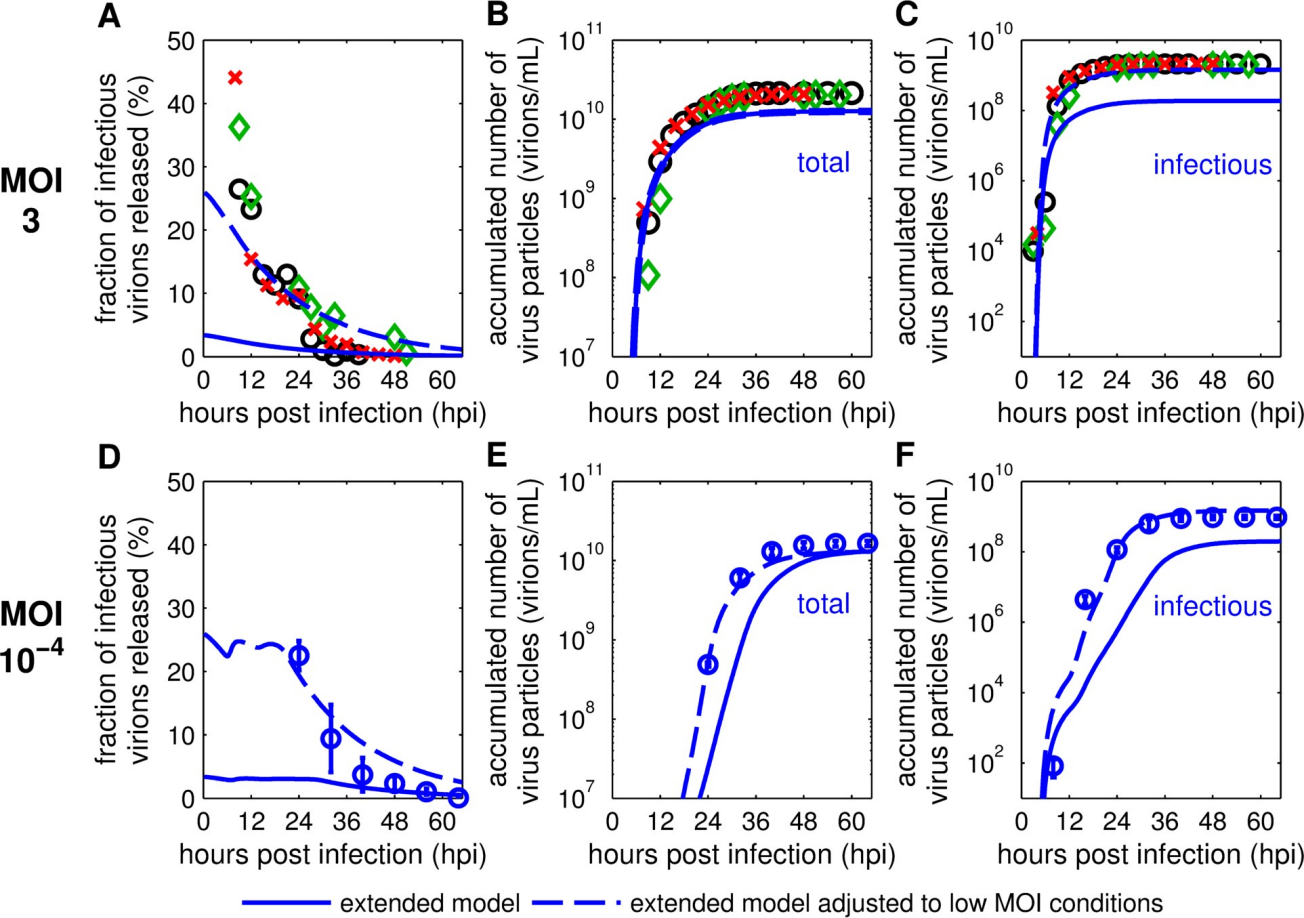
Model predictions of viral titers in low MOI conditions. The mathematical model fitted to measurements obtained from MDCK cell infections at an MOI of 73 (S1 Fig) was used to reproduce results of experiments performed at (A-C) MOI 3 and (D-F) MOI 10^−4^ based on TCID_50_. Symbols represent measurements of MDCK cell infections with influenza A/PR/8/34 (H1N1) obtained from NIBSC (A-C) and RKI (D-F). (A+D) Description of the fraction of infectious virions released with different initial conditions. Model prediction for total (B+E) and infectious virus particles (C+F) released. Error bars in (D-F) indicate standard deviations of three individual experiments. Solid lines depict simulations of the extended model with initial virus concentrations adapted to the respective MOI. Dashed lines represent simulations of the extended model with an additional change to the initial conditions to adjust for low MOI scenarios, i.e. by increasing the initial fraction of infectious virions released.

First, we simulated the infection dynamics of different MOI conditions by exclusively changing the initial amount of infecting virus particles. Unfortunately, this approach did not result in satisfying predictions (Fig 5, solid lines). While the total virus particle concentration for an infection at MOI 3 based on TCID_50_ could be described (Fig 5B), other measurements differed significantly from model simulations in both dynamics and magnitude of viral release. Additionally, we observed clear differences in the FIVR dynamics between simulations and low MOI measurements. In experiments performed at MOI 3 and 10^−4^ (based on TCID_50_), the FIVR initially shows considerably higher values (Fig 5A and 5D) than at MOI 73 (Fig 3A). This indicates that cells infected at lower MOIs have a significantly higher ratio of infectious to non-infectious virus particles released than cells infected at a high MOI.

Consequently, to improve the model prediction, we adjusted the initial FIVR *F*_Par_(0) to low MOI conditions. To only introduce a single new *F*_Par_(0) for the low MOI conditions we tested different values and their effect on model predictions for MOI 3 and 10^−4^ based on TCID_50_ (S5 Fig). We found an optimal value with *F*_Par_(0) = 0.26, which provided the best description of the FIVR for both low MOI conditions (Fig 5A and 5D). As a result, the model predictions for virus release using the adjusted initial condition are in good agreement with the two experiments performed at lower MOIs (Fig 5). In particular, the time delay before cells start to release considerable amounts of virus particles, which is heavily dependent on the MOI conditions, can be well described. Thus, by considering the influence of a critical initial condition, i.e. *F*_Par_(0), during influenza virus infection our model captures the viral release dynamics of both high and lower MOI infections in MDCK cell cultures.

**Fig 6:**
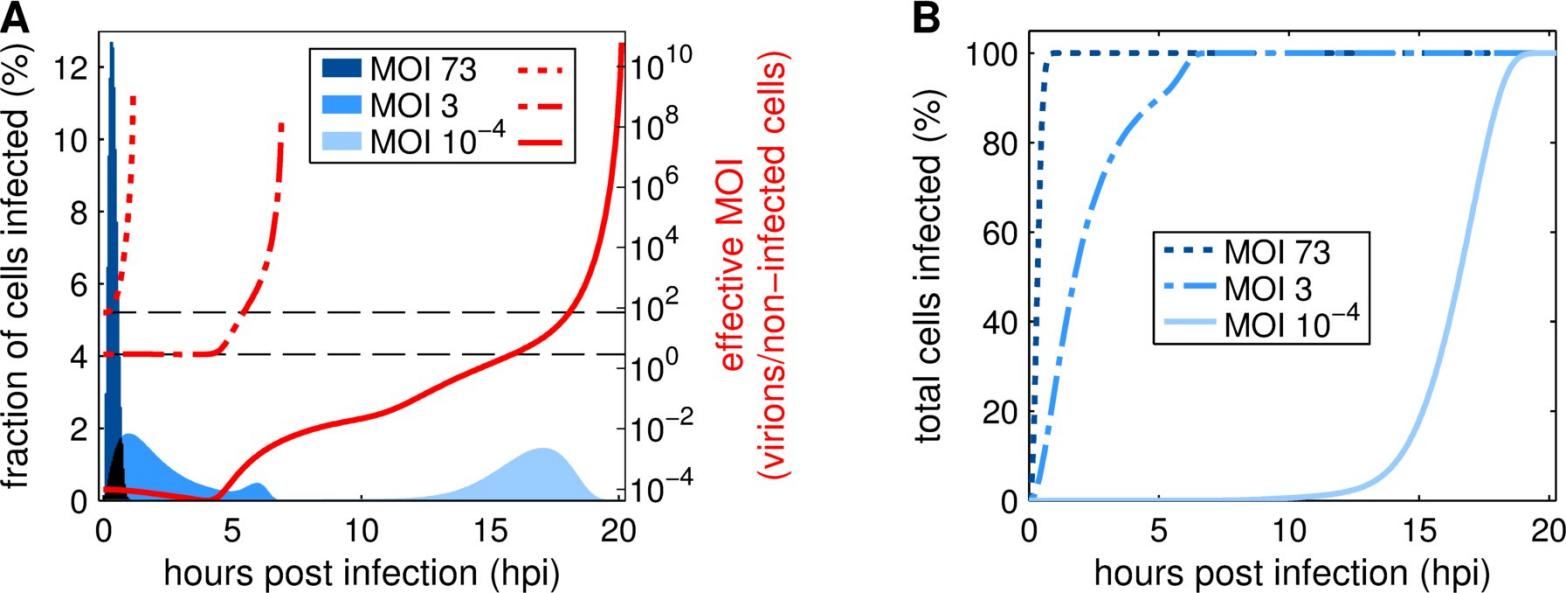
Progress of cell infection and effective MOI over the time course of IAV infection. Cell culture infections were simulated with initial MOIs of 73, 3 and 10^−4^ based on TCID_50_. (A) Bars depict the amount of cells newly infected in the respective time frame (1 bar = 0.05 h). Dotted, dash-dotted and solid lines represent the current effective MOI based on the number of infectious virus particles and the remaining non-infected cells in the culture. The horizontal dashed lines highlight effective MOIs of 3 and 73. (B) Curves show the cumulative frequency of cell infections for simulations performed at MOI 73, 3 and 10^−4^.

In addition to viral titers the model is able to predict various intra- and extracellular processes during the IAV infection of cell cultures in different MOI conditions. As an example, we simulated the fraction of cells infected and the ratio of infectious virions to non-infected cells (the effective MOI) over the time course of virus replication at MOIs of 73, 3 and 10^−4^ (Fig 6). The effective MOI can change considerably over the progress of infection and determines how many virions enter a cell at a specific time point influencing virus replication and release. In high MOI conditions, cells are infected rapidly until 1 hpi and the effective MOI increases instantly. Simulations with an MOI of 3 result in a slower progress of infection that shows two peaks indicating a second infection wave starting around 4 hpi. Here, the effective MOI stays constant up to 4 hpi and then increases until all cells are infected. Overall, most cells in infections performed at initial MOIs of 73 and 3 are infected at similar effective MOIs. The simulation of an infection performed at an initial MOI of 10^−4^ shows very different results. The infection progress is delayed considerably and the model prediction suggests that most cell infections occur not before 15 hpi. Additionally, the effective MOI decreases until 4 hpi and only then starts to increase gradually. The dynamics of the effective MOI progresses in multiple waves showing step-like behavior around 4, 11 and 17 hpi. This strong variation of the effective MOI during the process induces very different infection scenarios for cells infected at different time points. Additionally, the majority of cell infections, around 66%, occur at an effective MOI of 3 or higher, despite the low initial MOI. In summary, the extended model predicts a rapid, uniform infection in high MOI conditions and a delayed progress of infection with variations in the effective MOI (multiple waves) in a low MOI scenario.

## Discussion

The mechanisms of influenza virus infection are governed by highly complex processes including various virus-host cell interactions, an adaptive viral defense against cellular immune responses, and a multi-layered regulation of virus replication and spread. Novel experimental and computational methods enable in-depth investigations of these processes. Here, we present an augmented mathematical multiscale model of IAV infection that closely captures experimental data for low and high MOI infection conditions on the intra- and extracellular level. With this model, we successfully predict virus release dynamics and show how the MOI affects the progress of infection.

In contrast to the original multiscale model established by Heldt et al. [24], the extended model was calibrated to intra- and extracellular data from the same experiment [4]. The high MOI infection conditions used in these experiments enabled the measurement of dynamics on both cellular levels. We used these measurements for parameter estimation and the resulting model simulation showed good agreement with the experimental data (S1 Fig). Viral RNA dynamics and the percentage of cells in different states of infection and apoptosis were captured closely. However, between 3 and 8 hpi, model simulations underestimated the level of intracellular vRNA. Previous experimental studies also identified an accumulation of vRNA in this time frame [24,33], which cannot be fully reproduced by the current model implementation. The various viral RNA species of IAV are highly interconnected, because vRNA serves as the template for viral mRNA and cRNA replication. The underestimation of a single viral RNA species indicated that an additional layer of regulation, which supports earlier vRNA accumulation without impacting the other species, may exist. Such a regulation could concern nuclear export processes of viral proteins, the depletion of precursors for viral RNA and protein synthesis impeding further replication or an increased degradation of vRNPs that enter the cytoplasm for release. To test such hypotheses, experimental approaches could analyze the metabolism of infected cells, the stability of vRNPs in the nucleus and cytoplasm, or the availability of specific resources for virus particle synthesis, preferably in a high MOI scenario.

The most important step to reproduce infection dynamics in MDCK cell cultures at a high MOI was the description of apoptotic processes. In general, apoptosis is induced in infected cells as a defense mechanism aiming to reduce progeny virion release [34]. In infected cells, viral RNA and protein synthesis progress rapidly leading to an accumulation of viral molecules. This is detected by the cell and, as a reaction, apoptotic processes are induced that lead to controlled cell death to prevent further virus spread. In the original model [24], infected cell apoptosis was described by a fixed rate (Fig 2C), which resulted in an exponential distribution of the survival time of infected cells. However, experimental data of infected cells and their transition to an apoptotic state indicate a normal distribution of cell survival time (Fig 2A and 2B) [4], which was discussed previously by Holder et al. [25]. To accommodate these characteristics, we implemented a logistic function (Eq (9)) for the description of apoptosis induction in infected cells. Logistic functions can approximate the dynamics described by a normal distribution and are relatively simple to apply [26]. Other approximation functions exist, which can provide an even closer representation of the normal distribution. However, these functions are more complex than the logistic function and not required to describe the experimental data. This approximation of the survival time of infected cells enabled the reproduction of the apoptosis dynamics measured in high MOI influenza A infection experiments [4]. Additionally, by utilizing a logistic function, we introduced a delay before significant amounts of cells undergo apoptosis, which was experimentally observed until 16 hpi (Fig 2). This delay is represented by the newly introduced parameter *τ*_Apo_, which describes the time frame in which an infected cell induces apoptosis. Therefore, a low value for *τ*_Apo_ indicates that the cell can induce a fast response to the infection. However, viruses have developed mechanisms to interfere with the host cell apoptosis to prolong virus production [34]. Thus, the parameter *τ*_Apo_ could also show how well a virus is adapted to the host cell. The delayed apoptosis induction described in our extended model is similar to the cell death dynamics during HIV infection described in [35]. For the latter, a piecewise-defined function that includes a specific time delay was utilized to achieve an effect similar to the one resulting from the use of a logistic function in our model. Nevertheless, dynamics of influenza-induced cell death is highly strain-dependent [36], which could require different approaches based on the respective scenario.

For model calibration, we utilized accumulated virus titer measurements, which show the amount of virus particles produced since the previous sampling time point. Thus, the figures presented do not show virus degradation and the estimated rate of virus degradation, $k_{V}^{\mathrm{Deg}}$, is one order of magnitude lower (Table 2) than described previously [13]. Furthermore, this approach leads to a slight underestimation of the cell-specific yield of infectious virions, because infectious virus particle degradation is not taken into account. To enable a description of infectious virus titer measurements that were not determined cumulatively, the parameter for virus degradation should be re-estimated or taken from the related literature [13].

**Table 2:** Parameters estimated from the experimental data in S1 Fig.

Parameter	Value	Confidence interval (95%)^a^
*F*_Par_(0) (−)	3.4×10^−2^	(1.8–17.0)×10^−2^
$k_{T}^{\mathrm{Apo}}\;{(h}^{-1})$	7×10^−3^	(1.4–11.4)×10^−3^
$k_{M1}^{\mathrm{Bind}}\;{(\mathrm{molecules}}^{-1}\cdot h^{-1})$	9×10^−7^	(2.9–34.2)×10^−7^
$k_{M}^{\mathrm{Deg}}\;{(h}^{-1})$	0.33	0.12–1.35
$k_{V}^{\mathrm{Deg}}\;{(h}^{-1})$	1.15×10^−2^	(0.23–2.15)×10^−2^
*k*^Fus^ (h^−1^)	0.31	0.21–0.40
*K*_I_ (h^−1^)	0.11	0.08–0.29
*k*^Lys^ (h^−1^)	9.4×10^−3^	(1.9–35)×10^−3^
*K*_R_ (molecules)	1.1×10^7^	(0.1–5.7)×10^7^ ^b^
*k*^Rel^ (virions ⋅ h^−1^)	1270	917–2210
$k_{\mathrm{Red}}^{\mathrm{Rel}}\;{(h}^{-1})$	0.05	0.001–0.25^b^
$k_{C}^{\mathrm{Syn}}\;{(h}^{-1})$	0.8	0.4–1.7
$k_{M}^{\mathrm{Syn}}\;(\mathrm{nucleotides}\cdot h^{-1})$	1.8×10^5^	(0.9–3.4)×10^5^
$k_{V}^{\mathrm{Syn}}\;{(h}^{-1})$	8.4	5.2–12.9
$K_{V^{\mathrm{Rel}}}\;(\mathrm{virions})$	1250	92–4600
*τ*_Apo_ (h)	19.8	16.7–23.7
*ν*_Apo_ (h^−1^)	0.77	0.30–3.82

^a^ 95% confidence intervals were determined from the Q_0.025_ and Q_0.975_ quantiles of 2000 bootstrap iterations [32].

^b^ Estimates reached lower and upper bootstrap parameter bounds.

Infected cells release infectious and non-infectious progeny virions, which can be determined via TCID_50_ and HA assay results. The ratio of released infectious virions to the total amount of virus particles released, which is described by the introduced variable *F*_Par_ (Eq (4)), is a measure for the efficiency of IAV replication in cell culture. The highest FIVR occurs during early infection, which is crucial to enable fast virus spreading before host defense mechanisms, i.e. apoptotic processes or cellular immune response, may interfere. Over time, the FIVR decreases and during later stages of infection non-infectious particles are released predominantly (Fig 3A). A recent study [4] identified that during late infection (starting at around 20 hpi) the morphology of released particles changes, leading to more deformed or broken particles. This observation was linked to decreasing cell viability, the detachment of infected adherent cells and nuclear fragmentation indicating apoptotic processes in the infected cells. In addition, cellular metabolism is heavily affected by virus replication at later stages of infection [37]. Further investigations regarding the capability and bottlenecks of infected cells to release functional, infectious virions should be performed to determine the underlying mechanisms. Especially experiments that closer examine the composition of non-infectious particles, i.e. the fraction of DIPs, virions missing genomic information, and broken particles, in different MOI conditions would be valuable. This could advance the understanding of prerequisites for successful virus replication and release, which can support both vaccine production and antiviral strategies.

Furthermore, the FIVR differed significantly between low and high MOI cultivations. In low MOI conditions, a considerably increased FIVR was observed (Figs 3A, 5A and 5D). In particular, during experiments performed at an MOI of 10^−4^ based on TCID_50_, the FIVR maintained a value above 20% until 24 hpi. This dynamics can be explained by the continuous infection of cells until 20 hpi (Fig 6) which contributes with a high initial FIVR to the release characteristics of the infected cell population. Overall, the differences in viral release between low and high MOI infections have a significant impact on the infectious virus titer and the propagation of infections on the extracellular level (Fig 1). Although various factors influence the efficiency of virus replication and release, the presence of DIPs in high MOI seed virus and their accumulation over the time course of infection most likely play a key role regarding the observed disparity. Due to deletions in their genome, DIPs are non-infectious virus particles and, therefore, require the co-infection with an infectious virion for replication. During co-infection DIPs impair the replication of infectious virions, and co-infected cells predominantly release progeny DIPs [8]. In high MOI conditions nearly all cells are infected by more than one virus particle (S4 Fig) providing an optimal basis for DIP interference. Further support for a connection between the FIVR and DIP interference is provided by the correlation of DIP accumulation with the decrease of infectious virus particle release, which was observed in experiments performed at an MOI of 3 based on TCID_50_ (S2 Fig). However, the influence DIPs exert on the molecular level of virus replication is still not fully understood. Additional studies examining DIP interference dynamics and the FIVR could elucidate critical factors controlling virus spread dynamics in cell populations, e.g. by testing the behavior of such properties for a wide range of infection conditions.

MOI also heavily affects the progress of IAV infection in cell cultures. The higher the MOI, the more cells are infected and the earlier virus particles are released. Additionally, the initial MOI impacts the dynamics of the effective MOI resulting in highly different scenarios regarding the amount and the origin of infecting virions. In high MOI conditions, all cells are infected by the initially available virions in a single infection wave (Fig 6). In addition, such infections are with near certainty multiple-hit infections (S4 Fig). Simulations of an infection at MOI 3, however, show two different scenarios. Until 4 hpi cells are infected by infectious virions from the seed at an effective MOI that results in around 50% chance of inducing multiple-hit infections (S4 Fig). In a second infection wave, starting at 4 hpi, progeny virions begin to infect cells at increasing effective MOIs leading to a higher multiple-hit infection probability. Model predictions for low MOI conditions (10^−4^), which are applied typically in influenza vaccine production, show variations in the effective MOI that span 14 orders of magnitude (Fig 6). Using the MOI-sensitive modeling approach developed in this study enabled detailed analyses regarding such a scenario. Simulations show that effective MOI progresses in three infection waves starting at 0, 4 and 11 hpi. In the first 4 hpi seed virions infect target cells exclusively in single-hit infections. From 4 to 15 hpi first and second generation progeny virions induce additional infection waves, in which still mostly single-hit infections occur as the effective MOI stays below one (S4 Fig). Finally, the effective MOI increases steadily, so that 16 hpi mostly multiple-hit infections take place. Interestingly, over 64% of all cell infections occur after 16 hpi (Fig 6). Therefore, our model simulations predict that even in low MOI conditions the majority of cells are infected by multiple virions. Altogether, this indicates that cells in high MOI conditions are infected exclusively by the seed virus while in low MOI cultivations cells are infected almost exclusively by progeny virions. In both low and high MOI conditions mostly multiple-hit infections occur. However, during the initial phase of a low MOI infection predominantly single-hit infections take place. It would be valuable to assess these predictions by analyzing the dynamics of single- and multiple-hit infections in an infected cell culture using different initial MOIs.

Furthermore, the model predictions provide interesting implications for the interference of DIPs with infectious virions for different MOI conditions. In our simulations, the majority of IAV-infected cells are hit by multiple virions, regardless of the MOI. Such conditions favor DIP replication as they increase the chance of co-infections by DIPs and infectious virions. Therefore, it could be argued that even low MOI conditions do not prevent DIP interference, but only postpone it to later infection stages (Fig 6). However, there is an important distinction between different MOI conditions regarding the seed virus. In high MOI conditions all cells undergo multiple-hit infections by virions exclusively from the seed virus. Therefore, the amount of DIPs in the seed virus determines the severity of interference. To achieve a low impact of DIPs in high MOI conditions a “clean” seed virus with very low DIP content is required. This is in particular relevant for experimental studies aiming for single-step virus growth to avoid artifacts. But even for studies in small scale cultures or laboratory scale bioreactors, the quality of the seed virus should be controlled carefully to avoid misinterpretation of experimental findings. In low MOI conditions, relevant for cell culture-based influenza vaccine production, virions from the seed virus infect cells almost exclusively in single-hit infections preventing DIP interference and replication at early cultivation time. In later infection stages, progeny virions of the second to third generation nevertheless induce multiple-hit infections. Thus, the amount of DIPs generated *de novo* [18] during progeny virion production has a higher impact on the extent of interference with virus yields and the DIP content of the seed virus only plays a minor role. Altogether, DIPs always affect the replication of IAV regardless of the initial MOI. But, the interference has a lower impact and is postponed until later infection stages in low MOI conditions.

Another intriguing effect of different MOI conditions is their impact on viral mRNA dynamics. In high MOI conditions, viral mRNA accumulates rapidly (Fig 4A), reaches a distinct peak around 4 hpi, and declines thereafter. Various studies showed similar findings in high MOI IAV infections in different cell lines [27,33,38]. In contrast, low MOI scenarios induce a considerably slower accumulation with less pronounced peaks around 8 hpi [24,39,40]. The fast accumulation in high MOI infections is most likely induced by the increased amount of available templates (vRNA). The subsequent shutdown of viral mRNA synthesis is mediated by the export of vRNAs from the nucleus, which occurs around 4 hpi in high MOI experiments [4]. However, the first iteration of our extended model, which was modified by an adjusted apoptosis rate and the newly introduced FIVR, could not describe satisfactorily the shutdown of viral mRNA replication and its degradation (Fig 4). Model simulations showed a slow decrease of viral mRNA synthesis (Fig 4B), which was counteracted by a high mRNA degradation rate ($k_{M}^{Deg}=0.6$) to reproduce the drop of viral mRNA in the measurements. This rate is twice as high as a viral mRNA degradation rate determined previously [14]. Moreover, the dynamics of viral mRNA degradation is not captured fully (Fig 4A). Thus, we implemented an interaction between viral and host cell mechanisms that was reported recently by Rodriguez et al. [29] and Martínez-Alonso et al. [30]. They showed that the binding of free viral RdRp to cellular Pol II leads to the degradation of the latter, which is proposed as a method for inhibiting host gene expression. In addition, this mechanism would impede viral mRNA transcription, which depends on Pol II activity, and provides an explanation for the observed shutdown. After implementation of this interaction, the extended model is in good agreement with the viral mRNA dynamics during a high MOI infection (Fig 4A). Additionally, the viral mRNA degradation rate is now consistent with previous results ($k_{M}^{Deg}=0.3$). These findings indicate that the RdRp-mediated degradation of Pol II not only inhibits host gene expression, but also plays a role in the downregulation of the viral mRNA synthesis.

In summary, we adjusted an existing mathematical multiscale model of IAV infection in animal cell cultures [24]. In contrast to previous models, it explicitly considers the infection age of a cell regarding apoptotic processes and the release of infectious virus particles, which enables the description of both high and low MOI scenarios relevant for basic research and vaccine production. MOI-sensitive models could also benefit research on other viruses, e.g. plant viruses, in which the MOI is theorized to have an impact on virus evolution [41]. Furthermore, this model can be used to examine specific steps in the IAV life cycle in relation to the maximum virus yield or regarding measures to efficiently intervene with viral spread *in vivo*. Given available experimental data, future work could encompass a multiscale DIP replication model advancing the understanding of DIP impact on viral replication at different MOIs and characterizing DIP production for establishment of antiviral therapies [42]. Additionally, the model could be used to describe infections in tissues and organs at the within-host scale–a scenario in which the spatiotemporal MOI fluctuates strongly. To accomplish the abovementioned goals, the incorporation of DIP propagation, the host immune response and a model expansion to the second or third spatial dimension have to be considered. Ultimately, the predictive multiscale model presented here is well suited to predict and optimize process performance of IAV production in cell cultures and provides a solid framework for further analysis of MOI-dependent virus infections in general.

## Materials and methods

### Model of the intracellular level

The intracellular level of IAV infection is based on a model previously developed in our group [14]. In short, this model comprises a set of ordinary differential equations describing the essential steps of virus replication. These include virus entry, nuclear import, replication and transcription of viral RNA, protein synthesis, viral assembly and virus particle release (Eqs (S1)-(S30)). Additionally, the model was modified according to [24] by adjusting the viral release rate to

$$r^{\mathrm{Rel}}\left(\tau \right)=k^{\mathrm{Rel}}\frac{Vp_{M1}^{\mathrm{Cyt}}}{Vp_{M1}^{\mathrm{Cyt}}+8K_{V^{\mathrm{Rel}}}}\prod_{j}\frac{P_{j}}{P_{j}+N_{P_{j}}K_{V^{\mathrm{Rel}}}}$$ (1)

where j ∈ {RdRp, HA, NP, NA, M1, M2, NEP} and *τ* denotes the infection age of a cell. Viral release is determined by the available viral proteins, *P*_j_, and viral ribonucleoproteins (vRNPs) in the cytoplasm, $Vp_{M1}^{\mathrm{Cyt}}$. The parameters $N_{P_{j}}$ and $K_{V^{\mathrm{Rel}}}$ describe the number of viral proteins necessary for the formation of virus particles and the amount of viral components required to achieve half the maximum virus release rate, respectively. Based on this implementation, virus particle release is confined by the parameter *k*^Rel^, which defines a maximum release rate. For a detailed discussion of the intercellular dynamics the reader is referred to the original publications [14,24].

We extended this model by assuming that an infected cell releases both infectious and non-infectious virus particles. Hence, we considered *r*^Rel^ as the release rate of all virus particles after intracellular replication and introduced the FIVR *F*_Par_(*τ*) to define the release of infectious virus particles. In the resulting equations

$$r_{\mathrm{Par}}^{\mathrm{Rel}}(\tau )=r^{\mathrm{Rel}}(\tau )$$ (2)

$$r_{\mathrm{Inf}}^{\mathrm{Rel}}(\tau )=r^{\mathrm{Rel}}(\tau )F_{\mathrm{Par}}(\tau )$$ (3)

$r_{\mathrm{Par}}^{\mathrm{Rel}}(\tau )$ and $r_{\mathrm{Inf}}^{\mathrm{Rel}}(\tau )$ describe the infection age-dependent release rates for all virus particles and infectious virions by an individual cell, respectively. Experiments in our group revealed that *F*_Par_(*τ*) is decreasing over time [4]. Consequently, we defined it as a first order degradation

$$\frac{dF_{\mathrm{Par}}}{dt}=-k_{\mathrm{Red}}^{\mathrm{Rel}}F_{\mathrm{Par}}$$ (4)

with $k_{\mathrm{Red}}^{\mathrm{Rel}}$ describing the observed decrease of infectious virus particle release. As the understanding of factors influencing the capability of cells to produce infectious particles is still limited [4], we chose to combine such effects in one parameter, $k_{\mathrm{Red}}^{\mathrm{Rel}}$.

Furthermore, we introduced an additional step of viral RNA regulation, i.e. the inhibition of viral mRNA synthesis, suggested in [29,30]. The authors propose that free viral RdRp degrades cellular Pol II, which would impede viral mRNA synthesis. We implemented this interaction by adjusting the standard mRNA dynamics (Eq (S16)) to

$$\frac{dR_{i}^{M}}{dt}=\frac{k_{M}^{\mathrm{Syn}}Vp^{\mathrm{Nuc}}}{8L_{i}\left(1+\frac{P_{\mathrm{Rdrp}}}{K_{R}}\right)}-k_{M}^{\mathrm{Deg}}R_{i}^{M}$$ (5)

with *P*_RdRp_ as the concentration of free viral RdRp and *K*_R_ describing the amount of free RdRp that has to be available to reduce mRNA synthesis by half. Synthesis and degradation rates of viral mRNA are defined by $k_{M}^{\mathrm{Syn}}$ and $k_{M}^{\mathrm{Deg}}$, respectively. The amount of vRNPs in the nucleus, *Vp*^Nuc^, represents the available viral template and *L*_i_ describes the segment-specific length of viral mRNA. The implementation of an inhibitory term based on the concentration of free RdRp enables a close description of the mRNA dynamics observed in high MOI conditions [4,33] by the extended model (Fig 4A).

### Model of the extracellular level

The model of the extracellular level of IAV infection is based on conventional cell population balances and follows the approach introduced in [24]. In brief, a set of integro-partial differential equations is combined with a set of ordinary differential equations to describe extracellular interactions. These include an age-segregated infected cell population, the transition of uninfected cells to an infected or apoptotic state, cell growth and death, virion production as well as the attachment and endocytosis of virus particles to infect cells (Eqs (S31)-(S47)).

For the description of both infectious and total virus particles on the population level we adjusted the extracellular dynamics. The total amount of released virus particles was implemented as

$$\frac{dP_{\mathrm{tot}}^{\mathrm{Rel}}}{dt}=\int_{0}^{\infty }r_{\mathrm{Par}}^{\mathrm{Rel}}\left(\tau \right)I\left(t,\tau \right)d\tau$$ (6)

with *I*(*t*,*τ*) denoting the age-segregated infected cell population. To stay in line with the newly introduced release rates (Eqs (2) and (3)) the balance equation for infectious virions was reformulated as

$$\frac{dV}{dt}=\int_{0}^{\infty }r_{\mathrm{Inf}}^{\mathrm{Rel}}\left(\tau \right)I\left(t,\tau \right)d\tau -k_{V}^{\mathrm{Deg}}V+\sum_{n}\left[k_{n}^{\mathrm{Dis}}V_{n}^{\mathrm{Att}}-k_{c,n}^{\mathrm{Att}}B_{n}V\right]$$ (7)

with n ∈ {hi,lo}, $k_{n}^{\mathrm{Dis}}$ and $k_{c,n}^{\mathrm{Att}}$ defining the dissociation and association rates of infectious virus particles to uninfected cells, $k_{V}^{\mathrm{Deg}}$ as the rate of virion degradation, $V_{n}^{\mathrm{Att}}$ referring to virions attached to the cell surface and *B*_n_ as the amount of free binding sites on the cell surface. As the experimental data provide measurements for the accumulated virus particle release, we introduced an additional equation describing the total amount of released infectious virus particles

$$\frac{dV_{\mathrm{tot}}^{\mathrm{Rel}}}{dt}=\int_{0}^{\infty }r_{\mathrm{Inf}}^{\mathrm{Rel}}\left(\tau \right)I\left(t,\tau \right)d\tau ,$$ (8)

which disregards virus internalization and degradation.

The apoptosis dynamics of the original model [24] was adjusted to comply with a normal distribution of the survival time of infected cells as indicated by recent experimental data [4] (shown in Fig 2A). To this end, we employed a logistic function, which can be used to approximate dynamics induced by the cumulative density function of the normal distribution [26], to describe the infection age-dependent cell apoptosis rate

$$k_{I}^{\mathrm{Apo}}(\tau )=\frac{K_{I}}{1+\exp(-v_{\mathrm{Apo}}(\tau -\tau_{\mathrm{Apo}}))}$$ (9)

with *K*_I_ as the maximum virus-induced apoptosis rate, *τ*_Apo_ referring to the time after cell infection at which the rate of virus-induced apoptosis reaches its half-maximum and *ν*_Apo_ as a factor that describes the time required from cell infection until the full activation of the apoptosis mechanism. The parameter *τ*_Apo_ can be directly related to the mean μ of the normal distribution and *ν*_Apo_ can be converted to the standard deviation *σ* of the normal distribution by calculating

$$\sigma =\frac{1.62}{\nu_{\mathrm{Apo}}}.$$ (10)

Additionally, we tested Gompertz and Hill functions for the description of a delay in apoptosis induction (S3 Fig) as they can also approximate the cumulative density function of the normal distribution. Finally, we decided to implement a logistic function as it provides the best fit to our experimental data and enables a comprehensive adaptation to different scenarios including a step-like or smooth as well as an instant or delayed increase of the virus-induced apoptosis rate. For the purposes of this publication, a delayed and smooth rate increase of the infected cell apoptosis rate was utilized (Fig 2C).

### Simulation approach

Model simulation was handled according to [24]. In brief, the extra- and intracellular models were decoupled to reduce the necessary computational effort. To that end, we assumed that the intracellular dynamics in our model are independent of extracellular events and the time that passed since the cell culture was infected. The dynamics on the extracellular level are, however, dependent on the virus particle release on the intracellular level. The two levels are linked by utilizing a reduced version of the intracellular model that neglects virus entry. This process is handled instead on the extracellular level by Eqs (S43)-(S47). Thus, for model simulations, we first evaluated dynamics of the intracellular model to determine $r_{\mathrm{Inf}}^{\mathrm{Rel}}(\tau )$ and $r_{\mathrm{Par}}^{\mathrm{Rel}}(\tau )$. Then, the calculated viral release rates were applied in Eqs (6)–(8) to simulate the extracellular dynamics.

The equations of the intracellular model (Eqs (S1)-(S30)) were solved numerically with the CVODE routine from SUNDIALS [43] on a Linux-based system. Model files and experimental data were processed with the Systems Biology Toolbox 2 [44] for MATLAB (version 8.0.0.783 R2012b, TheMathWorks Inc.). The extracellular model (Eqs (S31) and (S33)-(S47)) was solved with Euler's method at a step size of *dt* = 0.05 h. In Eqs (S35), (S36), (S38), (S41) and (S42), the integrals were calculated by using Eq (S37) in place of *I*(*t*,*τ*) and applying the rectangle method to approximate results.

### Parameter estimation and model prediction

Model parameters were estimated by simultaneously fitting the intra- and extracellular model to the respective set of experimental data [4]. Measurements consisted of viral RNA dynamics (Fig 4, S1 Fig) on the intracellular level as well as cell population dynamics and virus titers (Figs 2 and 3) on the extracellular level. The reduced intracellular model, which is used to calculate the extracellular dynamics, utilized the same parameters as the complete intracellular model. To determine an optimal set of parameters, the global optimization algorithm fSSm for solving nonlinear problems [45] was employed. Individual estimation steps were evaluated by normalizing errors to their corresponding maximum measurement value. Then, the sum of errors for the intra- and extracellular level were divided by the respective number of measurements and finally combined to represent the overall goodness of fit. The first measurement value was applied as an offset to the simulated values of viral RNA to match a background signal observed in the real-time RT-qPCR experiments. Confidence intervals in Table 2 were determined by bootstrapping [32] based on the standard deviations obtained from three independent experiments (S1 Fig).

In S1 Table initial conditions for the parameter fit and model prediction are presented. The initial amount of virus particles in the intracellular model *V*^Ex^(0) is based on the respective MOI with a minimum of one virion infecting a cell. Accordingly, the reduced intracellular model, which neglects virus entry, is initiated with

$$Vp^{\mathrm{Cyt}}(0)=8\frac{\mathrm{molecules}}{\mathrm{virion}}F_{\mathrm{Fus}}V^{\mathrm{Ex}}(0)$$ (11)

where *F*_Fus_ denotes the fraction of fusion-competent virions. The expression *Vp*^Cyt^(0) in the reduced intracellular model represents the amount of vRNPs that reach the cytoplasm after *V*^Ex^(0) virus particles have infected a cell. We assume that at least one full set of vRNP infects a cell resulting in a minimum of *Vp*^Cyt^(0) = 8 molecules. All parameter values for the intra- and extracellular model are shown in S2 and S3 Tables, respectively.

Predictions of viral release in low MOI conditions (Fig 5) were performed by simulating the model with the parameters fitted for high MOI experimental data [4]. The initial FIVR was changed as newly collected measurements revealed significant differences between the percentages of infectious virus particles among the total release in high and low MOI conditions (Figs 3A, 5A and 5D). For simulations at MOI 10^−4^ and 3 we applied the same initial FIVR, which was determined by testing a range of values from 0 to 1 to find an optimum for both conditions (S5 Fig).

### Cells and viruses

The adherent MDCK cells (ECACC, No. 84121903) used in infection experiments were grown in Glasgow’s minimum essential medium (GMEM) supplemented with 10% (v/v) fetal calf serum and 1% (v/v) peptone at 37°C and 5% CO_2_ atmosphere. The serum-free infection medium was composed of GMEM, 1% (v/v) peptone and trypsin (5 BAEE U/mL, Sigma-Aldrich, # T7409). For the infections at MOI 3 and MOI 73, the influenza virus strain A/Puerto Rico/8/34 (PR8) obtained from the National Institute for Biological Standards and Control (NIBSC) was utilized. The infection at an MOI of 10^−4^ was performed with a PR8 strain from the Robert Koch Institute (RKI). The seed virus titers were determined by TCID_50_ assay [46] as 1.29 × 10^9^ virions/mL for the NIBSC and 1.1 × 10^9^ virions/mL for the RKI strain.

### Virus infection and quantification

The virus infections at all MOIs were conducted according to the protocol in [4]. In the low MOI experiments, cells were infected at an MOI of 3 and 10^−4^ based on TCID_50_. To achieve high MOI conditions, infections were performed at 10 PFU/cell [4], which correlated to an MOI of about 73 based on TCID_50_. Confluent cells in T75 flasks were washed twice with phosphate-buffered saline (PBS) and infected at an MOI of 10^−4^, 3 or 73 in 3 mL infection medium for 1 h. The flasks were rocked every 20 min to maintain an even virus distribution and kept at 37°C in a 5% CO_2_ atmosphere. Then, the inoculum was removed and the cells were washed twice with PBS. For the analysis of viral RNA dynamics (real-time RT-qPCR) and the fraction of cells in different infection states (imaging flow cytometry), performed at MOI 73, 13 mL of infection medium was added to the flasks. One individual flask was used for assaying every sampling time point. For a detailed description of the real-time RT-qPCR and imaging flow cytometry procedures the reader is referred to [4].

To analyze viral release kinetics, 5 mL infection medium was added after washing. Every 4 h, the supernatant was harvested. After removal of the supernatant, another 5 mL infection medium was used to wash the cells and added to the harvest. Finally, the flask was resuspended with 5 mL fresh infection medium for further incubation. Samples of the harvest were stored at -80°C until further analysis. Infectious and total viral titers were determined by TCID_50_ assay [46] and HA assay [47], respectively. The total virus particle titer is measured as log_10_ HA units per test volume (log HAU/100 μL). By assuming that the number of erythrocytes (2 × 10^7^ cells/mL) used in this assay is directly proportional to the number of virus particles required for agglutination, the concentration of hemagglutinating particles per mL is given by [48]:

$$c_{\mathrm{Virus}}=2\cdot 10^{7}\cdot 10^{\left(\log\;\mathrm{HAU}/100\;\mu L\right)}$$ (12)

## Supporting information

S1 Appendix Full list of equations for the multiscale model. (DOCX)

S1 Data Experimental data used for model calibration and prediction. (XLS)

S1 Fig The extended multiscale model captures intra- and extracellular infection dynamics. Curves depict the fits of the original and extended model to (A-C) cell-specific viral RNA, (D) cell population and (E) virus titer measurements obtained in MDCK cell-culture infections with influenza A/PR/8/34 (H1N1) at an MOI of 73 based on TCID_50_ [4]. Symbols represent the mean and error bars the standard deviation of three independent experiments. The extended model includes an adjusted apoptosis rate and takes into account the fraction of infectious virions released as well as an additional mechanism for inhibition of viral mRNA synthesis.
(TIF)

S2 Fig Correlation of the accumulation of DIPs and the reduction of infectious virus particles released. (A) Percentage of infectious virus particles released compared to the total number of virions released based on TCID_50_ and HA assay results. Time course data of three individual experiments for an infection at MOI 3 are shown. (B) Samples of one time series (A, circles) were analyzed via segment-specific RT-PCR to reveal intracellular accumulation of viral RNAs. For segment 1 full-length (FL) and defective interfering (DI) RNAs are depicted. Segment 5 FL RNA is shown as a control.
(TIF)

S3 Fig Different implementations of the rate function used to describe virus-induced apoptosis. Model fits to cell population measurements of (A) infected, non-apoptotic and (B) infected, apoptotic cells. Infection experiments were performed with MDCK cell cultures using influenza A/PR/8/34 (H1N1) at an MOI of 73 based on TCID_50_ [4]. Mean values of imaging flow cytometry results of three independent experiments are shown.
(TIF)

S4 Fig The chance of multiple-hit infections is determined by the effective MOI. Simulation of the probability that a cell is infected by more than one virion depending on the effective MOI. Calculations are based on the Poisson distribution. Dashed vertical lines indicate an effective MOI of 3 and 73, respectively.
(TIF)

S5 Fig Optimization of the initial fraction of infectious virions released in low MOI conditions. Simulation of the extended model with an MOI of (A) 3 and (B) 10^−4^ based on TCID_50_ using different initial FIVRs. Various initial FIVRs were tested for their ability to improve the model prediction for virus release dynamics in low MOI infections. Simulation results were evaluated based on their deviation to the experimental data and showed different optima at MOI 3 (*F*_Par_(0) = 0.46) and MOI 10^−4^ (*F*_Par_(0) = 0.21). The shared optimum (*F*_Par_(0) = 0.26) was determined by summing up deviations of both MOI 3 and 10^−4^ to obtain the initial FIVR resulting in the lowest error.
(TIF)

S1 Methods Segment-specific RT-PCR. (DOCX)

S1 Table Initial conditions for the extended multiscale model. (DOCX)

S2 Table Parameters of the intracellular model. (DOCX)

S3 Table Parameters of the extracellular model. (DOCX)
