# Supplementary material for: Multiscale modeling of influenza A virus replication in cell cultures predicts infection dynamics for highly different infection conditions
Source: PLoS Comput Biol. 2019 Feb 19;15(2):e1006819. doi: 10.1371/journal.pcbi.1006819 (PMC6396949; doi:10.1371/journal.pcbi.1006819)
Supplement: S1 Appendix — (DOCX) [file pcbi.1006819.s001.docx]

**S1 Appendix. Full list of equations for the multiscale model.**

The model equations applied in this publication and a description of the used variables is presented in this appendix. A description of model parameters is provided in S2 and S3 Table.

**Intracellular model**

The intracellular model is based on a model developed in [1]. Eq (S17) and Eq (S28)-(S30) were introduced or modified for this publication.

**Virus entry**

$$\frac{dV^{\text{Ex}}}{dt}=k_{Hi}^{\text{Dis}}V_{\text{Hi}}^{\text{Att}}+k_{\text{Lo}}^{\text{Dis}}V_{\text{Lo}}^{\text{Att}}-\left(k_{\text{Hi}}^{\text{Att}}B_{\text{Hi}}+k_{\text{Lo}}^{\text{Att}}B_{\text{Lo}}\right)V^{\text{Ex}}$$

with $B_{\text{n}}=B_{\text{n}}^{\text{Tot}}-V_{\text{n}}^{\text{Att}},\text{ n}\in\left\{\text{Hi},\text{Lo}\right\}$

with $k_{\text{n}}^{\text{Dis}}=\frac{k_{\text{n}}^{\text{Att}}}{k_{\text{n}}^{\text{Eq}}},\text{ n}\in\left\{\text{Hi,Lo}\right\}$

and $k_{\text{Ven}}^{\text{Deg}}=\frac{1-F_{\text{Fus}}}{F_{\text{Fus}}}k^{\text{Fus}},\text{ }0<F_{\text{Fus}}\leq1$

$V^{\text{Ex}}$ denotes extracellular virus particles that bind to free binding sites $B_{\text{n}}$ (sialic acid residues) on the cell membrane. Based on [4], we differentiate high-affinity (n = hi) and low-affinity (n = lo) binding sites. Virus particles attached to the cell surface ($V_{\text{n}}^{\text{Att}}$) can either dissociate from the binding sites with rate $k_{\text{n}}^{\text{Dis}}$ or perform a receptor-mediated endocytosis. Then, the enveloped virus particles ($V^{\text{En}}$) either fuse with the endosomal membrane to release their genome segments into the cytoplasm or are degraded in lysosomes if they are not capable of performing the fusion.

**Virus replication**

After the viral ribonucleoproteins (vRNP [$Vp^{\text{Cyt}}$]) reach the cytoplasm they can enter the nucleus. There, the vRNPs act as templates for viral replication ($Vp^{\text{Nuc}}$). Complementary RNA (cRNA [$R^{\text{C}}$]) is transcribed from the templates and then stabilized by binding viral RNA-dependent-RNA-polymerase (RdRp [$P_{\text{RdRp}}$]) to form $R_{\text{RdRp}}^{\text{C}}$ and binding nucleoproteins (NP [$P_{\text{NP}}$] ) to form complementary ribonucleoproteins (cRNP [$Cp$]). The naked cRNA is highly susceptible to degradation, but due to the stabilization it is degraded by lower rates ($k_{\text{R}}^{\text{Deg}}$ > $k_{\text{RRdRp}}^{\text{Deg}}$ > $k_{\text{Rnp}}^{\text{Deg}}$). The cRNP is the template for intracellular viral RNA (vRNA) transcription. Similar to the cRNA, the newly formed vRNA ($R^{\text{V}}$) is stabilized by binding RdRp and NP forming $R_{\text{RdRp}}^{\text{V}}$and $Vp^{\text{Nuc}}$, respectively.

To prepare the vRNP for the nuclear export, it is binding matrix protein 1 (M1) to form $Vp_{\text{M1}}^{\text{Nuc}}$. This complex is replication incompetent, but can leave the nucleus after binding to nuclear export protein (NEP [$P_{\text{NEP}}$]). When the vRNP carrying M1 is exported from the nucleus, it is denoted as $Vp_{\text{M1}}^{\text{Cyt}}$ and travels to the cell membrane.

**Viral transcription and protein synthesis**

The Eq (S16) and (S17) describe different realizations of the viral mRNA dynamics. In the original models [1,2] and for the simulation of the extended model without inhibition of viral mRNA synthesis (Fig 4, dotted line) the standard implementation described in Eq (S16) was used. In the extended model Eq (S17) was implemented.

original model: $\frac{dR_{\text{i}}^{\text{M}}}{dt}=\frac{k_{\text{M}}^{\text{Syn}}Vp^{\text{Nuc}}}{8L_{\text{i}}}-k_{\text{M}}^{\text{Deg}}R_{\text{i}}^{\text{M}}$

extended model: $\frac{dR_{\text{i}}^{\text{M}}}{dt}=\frac{k_{\text{M}}^{\text{Syn}}Vp^{\text{Nuc}}}{8L_{\text{i}}\left(1+\frac{P_{\text{Rdrp}}}{K_{\text{R}}}\right)}-k_{\text{M}}^{\text{Deg}}R_{\text{i}}^{\text{M}}$

Inside the nucleus, vRNPs also transcribe viral mRNA ($R_{\text{i}}^{\text{M}}$) which is necessary for viral protein synthesis. Each of the eight genome segments i encodes for different viral mRNAs. We assume that the transcription of viral mRNAs is dependent on their respective length ($L_{\text{i}}$) and that they are degraded with the rate $k_{\text{M}}^{\text{Deg}}$. In the extended model we introduce an additional regulation that reduces viral mRNA synthesis depending on the availability of free RdRp (Eq (S17)). Viral mRNA is translated into viral proteins $P_{\text{i}}$ in the cytoplasm. The three polymerase subunits ($P_{\text{PB1}}$, $P_{\text{PB2}}$ and $P_{\text{PA}}$) unite to form the RdRp, which is essential for virus replication. Genome segment seven encodes for both M1 and M2 and the fraction of the produced mRNAs for the respective protein is defined by the parameter $F_{\text{Spl7}}$. Besides their role in viral replication, the synthesized proteins are also required for virion release as they perform important structural functions in the virus particle, e.g. as surface proteins or ion channels.

**Virus particle release**

with $\text{j}\in\left\{\text{RdRp,HA,NP,NA,M1,M2,NEP}\right\}$

$$\frac{dP^{\text{Rel}}}{dt}=r_{\text{Par}}^{\text{Rel}}(\tau)=k^{\text{Rel}}\frac{Vp_{\text{M1}}^{\text{Cyt}}}{Vp_{\text{M1}}^{\text{Cyt}}+8K_{\text{V}^{\text{Rel}}}}\prod_{\text{j}} \frac{P_{\text{j}}}{P_{\text{j}}+N_{P_{\text{j}}}K_{\text{V}^{\text{Rel}}}}$$

The processes of virus particle assembly and release are implemented as a single step. To that end, the abundance of required viral proteins and progeny vRNPs is considered for the release of infectious virions ($V^{\text{Rel}}$) and the total amount of virus particles released ($P^{\text{Rel}}$). The percentage of infectious virions that are produced is determined by the variable $F_{\text{Par}}$, which decreases over time of infection.

**Extracellular model**

The extracellular model is based on the standard cell population balance with an expansion introduced in [2] to adjust it for IAV infection of cell cultures. Eq (S33), (S38) and (S41)-(S42) were introduced or modified for this publication.

**Cell populations**

with $k_{\text{I}}^{\text{Apo}}(\tau)=\frac{K_{\text{I}}}{1+\exp(-v_{\text{Apo}}(\tau-\tau_{\text{Apo}}))}$

with $\mu=\left[\frac{\mu_{\text{Max}}}{T_{\text{Max}}}\left(T_{\text{Max}}-T-\int_{0}^{\infty} I\left(t,\tau\right)\text{ }d\tau\right)\right]_{+}$

The extracellular model describes populations of uninfected cells (*T*), apoptotic uninfected cells (*T*_A_), infected cells (*I*) and apoptotic infected cells (*I*_A_). Uninfected cells can grow with rate *μ*, get infected with rate $r^{\text{Inf}}$ and undergo apoptosis with rate $k_{\text{T}}^{\text{Apo}}$. Infected cells are additionally affected by the virus-induced apoptosis rate $k_{\text{I}}^{\text{Apo}}(\tau)$ that is dependent on the infection age *τ*. Apoptotic cells are lysed with the rate $k_{\text{Lys}}$. The age-segregated population of infected cells $I(t,\tau)$ in Eq (S37) is classified by the infection age *τ* and considers how cells infected at time *t* – *τ* are affected by apoptosis.

**Virus particle release**

$$\frac{dV}{dt}=\int_{0}^{\infty} r_{\text{Inf}}^{\text{Rel}}\left(\tau\right)I\left(t,\tau\right)d\tau-k_{\text{V}}^{\text{Deg}}V+\sum_{\text{n}} \left[k_{\text{n}}^{\text{Dis}}V_{\text{n}}^{\text{Att}}-k_{\text{c,n}}^{\text{Att}}B_{\text{n}}V\right],$$

with $B_{\text{n}}=B_{\text{n}}^{\text{Tot}}\left(T+T_{\text{A}}\right)-V_{\text{n}}^{\text{Att}}$ ,

and $k_{\text{n}}^{\text{Dis}}=\frac{k_{\text{c,n}}^{\text{Att}}}{k_{\text{c,n}}^{\text{Eq}}}\text{ , n}\in\left\{\text{Hi,Lo}\right\}$

Infectious virus particles (*V*) in the extracellular space attach to high- and low affinity receptors $B_{\text{n}}$ on uninfected cells and can get degraded over time with the rate $k_{\text{V}}^{\text{Deg}}$. Infected cells produce these virions with the age-dependent release rate $r_{\text{Inf}}^{\text{Rel}}(\tau)$. Furthermore, the total amount of virus particles released, i.e. infectious and non-infectious particles, is described by the variable $P_{\text{tot}}^{\text{Rel}}$. To represent the cumulative infectious virus titer, which was measured in [3], we additionally introduced the variable $V_{\text{tot}}^{\text{Rel}}$.

**Virus entry**

with

The entry of virus particles into an uninfected cell is also described on the extracellular level, because it is linked to a reduced intracellular model to simulate both infection levels. Free infectious virions attach to and dissociate from cells with the rates $k_{\text{c,n}}^{\text{Att}}$ and $k_{\text{n}}^{\text{Dis}}$, respectively. Then, attached virus particles $V_{\text{n}}^{\text{Att}}$ can perform receptor-mediated endocytosis with the rate $k^{\text{En}}$. The additional loss of attached virions on the extracellular level due to infection or cell lysis occurs with the rates $r^{\text{Inf}}$ and $r^{\text{Lys}}$. These two rates are calculated based on the amount of virions in endosomes $V^{\text{En}}$ and apoptotic cells *T*_A_ in relation to the total amount of uninfected cells (*T* + *T*_A_), respectively.

**Supplementary references**

1. Heldt FS, Frensing T, Reichl U. Modeling the intracellular dynamics of influenza virus replication to understand the control of viral RNA synthesis. Journal of Virology. 2012;86(15): 7806-7817.
2. Heldt FS, Frensing T, Pflugmacher A, Gröpler R, Peschel B, Reichl U. Multiscale modeling of influenza A virus infection supports the development of direct-acting antivirals. PLoS Computational Biology. 2013;9(11): e1003372.
3. Frensing T, Kupke SY, Bachmann M, Fritzsche S, Gallo-Ramirez LE, Reichl U. Influenza virus intracellular replication dynamics, release kinetics, and particle morphology during propagation in MDCK cells. Applied Microbiology and Biotechnology. 2016;100(16):7181-7192.
4. Nunes-Correia I, Ramalho-Santos J, Nir S, de Lima MCP. Interactions of influenza virus with cultured cells: Detailed kinetic modeling of binding and endocytosis. Biochemistry. 1999;38(3): 1095-1101.
5. Arava Y, Wang YL, Storey JD, Liu CL, Brown PO, Herschlag D. Genome-wide analysis of mRNA translation profiles in Saccharomyces cerevisiae. Proceedings of the National Academy of Sciences of the United States of America. 2003;100: 3889-3894.
6. Robb NC, Jackson D, Vreede FT, Fodor E. Splicing of influenza A virus NS1 mRNA is independent of the viral NS1 protein. Journal of General Virology. 2010;91: 2331-2340.
7. Amorim MJ, Bruce EA, Read EKC, Foeglein A, Mahen R, Stuart AD, et al. A Rab11-and Microtubule-Dependent Mechanism for Cytoplasmic Transport of Influenza A Virus Viral RNA. Journal of Virology. 2011;85: 4143-4156.
8. Babcock HP, Chen C, Zhuang XW. Using single-particle tracking to study nuclear trafficking of viral genes. Biophysical Journal. 2004;87: 2749-2758.
9. Spirin, AS. Ribosome structure and protein biosynthesis. The Benjamin/Cummings Publishing Company. 1986
10. Lamb RA, Krug RM. Orthomyxoviridae: the viruses and their replication. In: Knipe DM, Howley PM, Griffin EG, editors. Fields virology, 4th edition. Lippincott Williams & Wilkins; 2001. p.1487-1531
11. Wakefield L, Brownlee GG. Rna-Binding Properties of Influenza-a Virus Matrix Protein M1. Nucleic Acids Research. 1989;17: 8569-8580.
12. Portela A, Digard P. The influenza virus nucleoprotein: a multifunctional RNA-binding protein pivotal to virus replication. Journal of General Virology. 2002;83: 723-734.
13. Schulze-Horsel J, Schulze M, Agalaridis G, Genzel Y, Reichl U. Infection dynamics and virus-induced apoptosis in cell culture-based influenza vaccine production-Flow cytometry and mathematical modeling. Vaccine. 2009;27: 2712-2722.
14. Frensing T, Pflugmacher A, Bachmann M, Peschel B, Reichl U. Impact of defective interfering particles on virus replication and antiviral host response in cell culture-based influenza vaccine production. Applied Microbiology and Biotechnology. 2014;98:8999-9008.
