# Supplementary figures and images for: Multiscale modeling of influenza A virus replication in cell cultures predicts infection dynamics for highly different infection conditions
Source: PLoS Comput Biol. 2019 Feb 19;15(2):e1006819. doi: 10.1371/journal.pcbi.1006819 (PMC6396949; doi:10.1371/journal.pcbi.1006819)

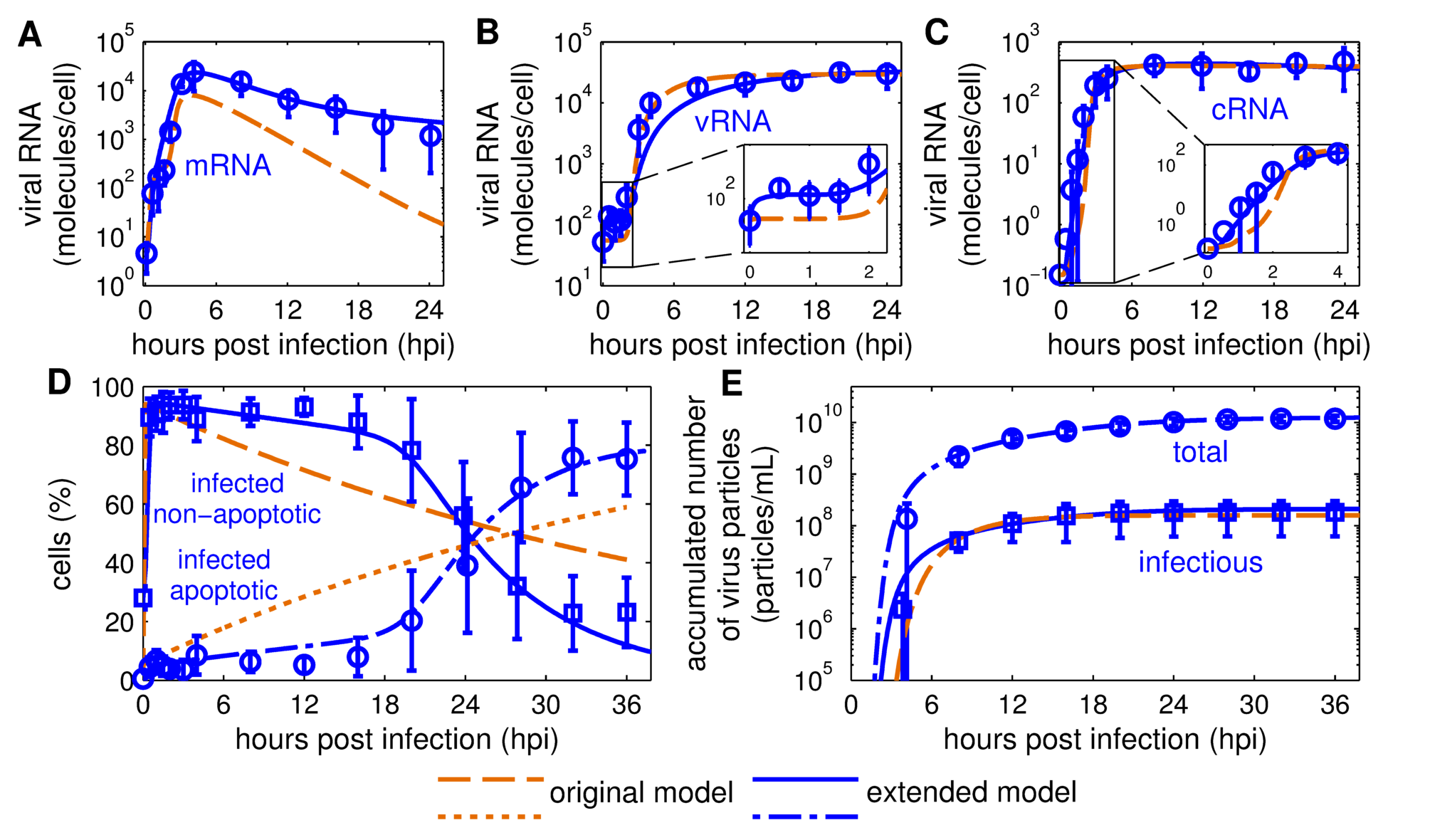

Supplement: S1 Fig — Curves depict the fits of the original and extended model to (A-C) cell-specific viral RNA, (D) cell population and (E) virus titer measurements obtained in MDCK cell-culture infections with influenza A/PR/8/34 (H1N1) at an MOI of 73 based on TCID50 [4]. Symbols represent the mean and error bars the standard deviation of three independent experiments. The extended model includes an adjusted apoptosis rate and takes into account the fraction of infectious virions released as well as an additional mechanism for inhibition of viral mRNA synthesis. (TIF) [file pcbi.1006819.s003.tif]

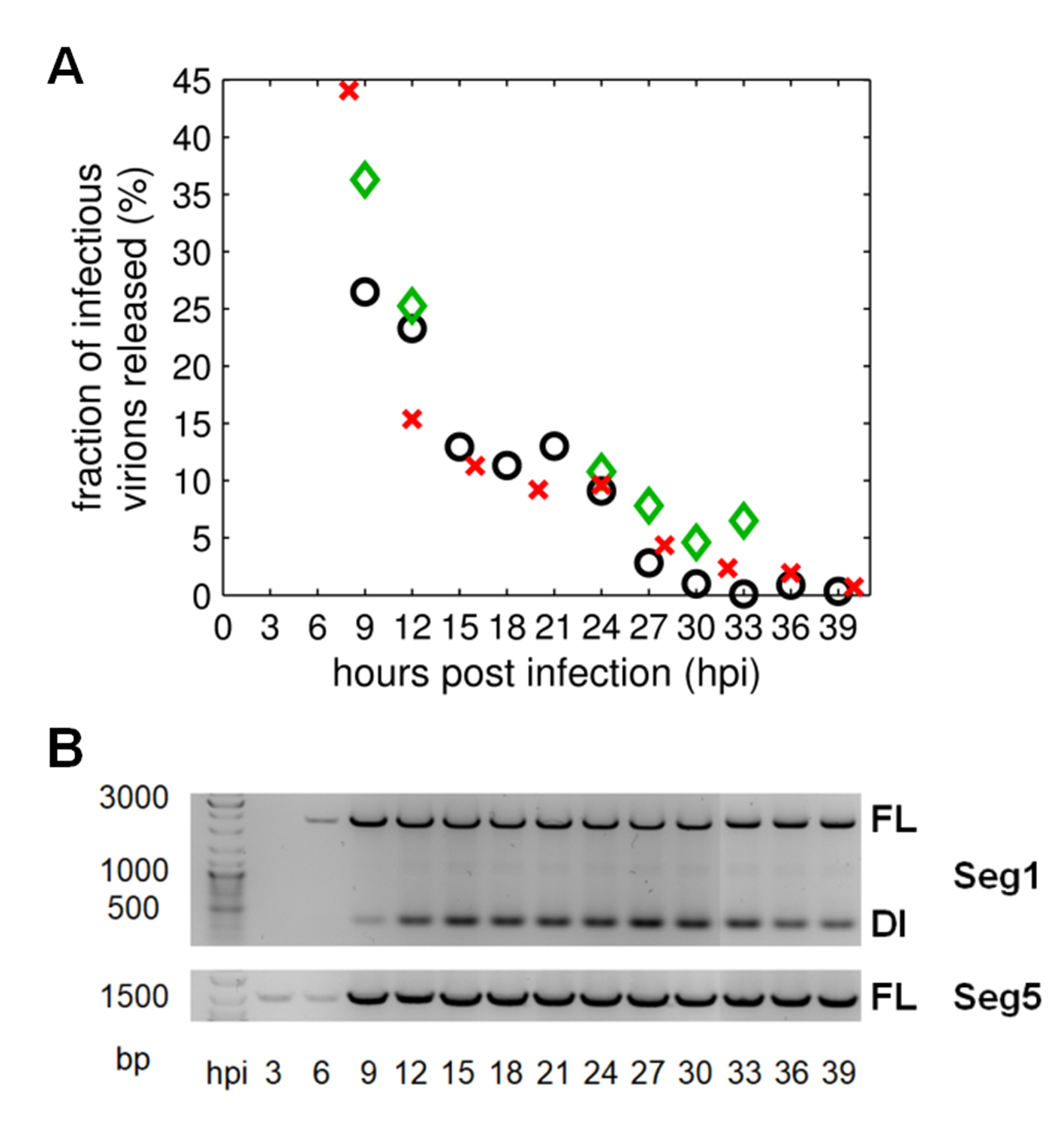

Supplement: S2 Fig — (A) Percentage of infectious virus particles released compared to the total number of virions released based on TCID50 and HA assay results. Time course data of three individual experiments for an infection at MOI 3 are shown. (B) Samples of one time series (A, circles) were analyzed via segment-specific RT-PCR to reveal intracellular accumulation of viral RNAs. For segment 1 full-length (FL) and defective interfering (DI) RNAs are depicted. Segment 5 FL RNA is shown as a control. (TIF) [file pcbi.1006819.s004.tif]

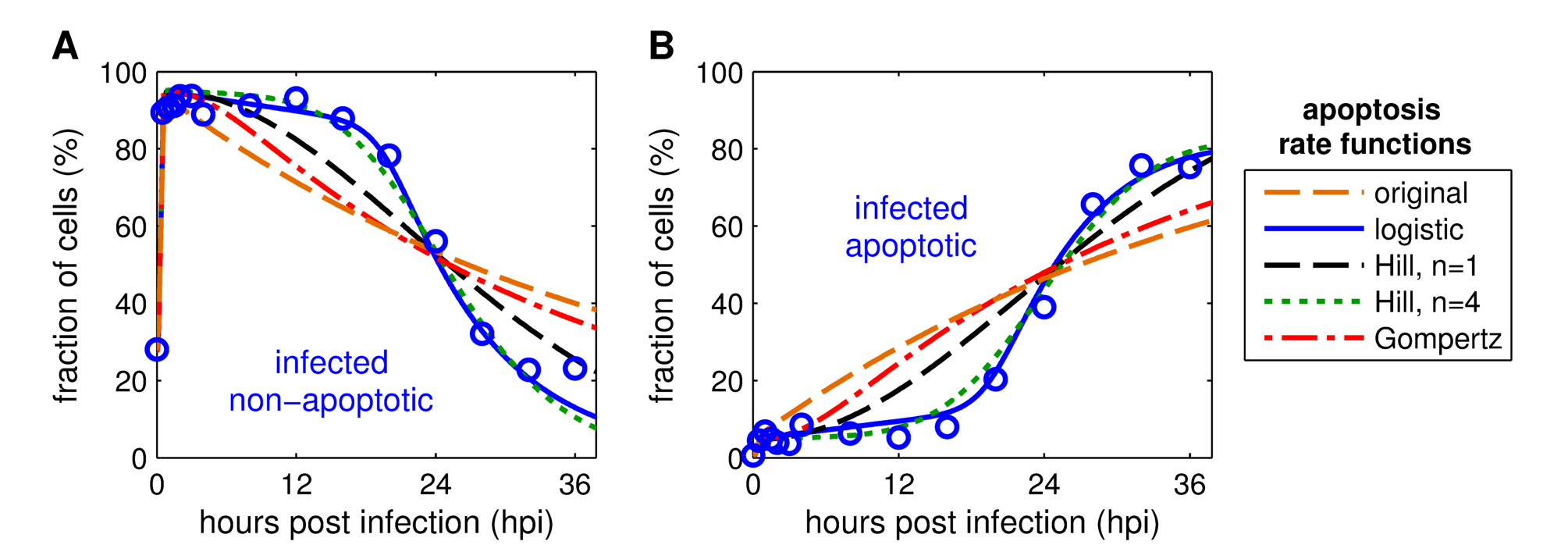

Supplement: S3 Fig — Model fits to cell population measurements of (A) infected, non-apoptotic and (B) infected, apoptotic cells. Infection experiments were performed with MDCK cell cultures using influenza A/PR/8/34 (H1N1) at an MOI of 73 based on TCID50 [4]. Mean values of imaging flow cytometry results of three independent experiments are shown. (TIF) [file pcbi.1006819.s005.tif]

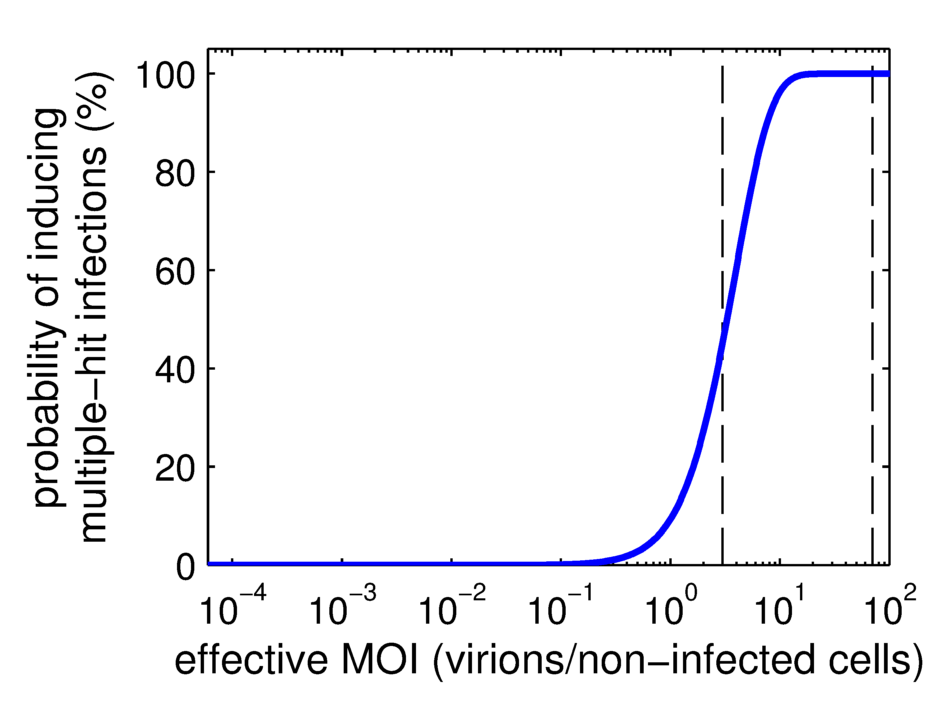

Supplement: S4 Fig — Simulation of the probability that a cell is infected by more than one virion depending on the effective MOI. Calculations are based on the Poisson distribution. Dashed vertical lines indicate an effective MOI of 3 and 73, respectively. (TIF) [file pcbi.1006819.s006.tif]

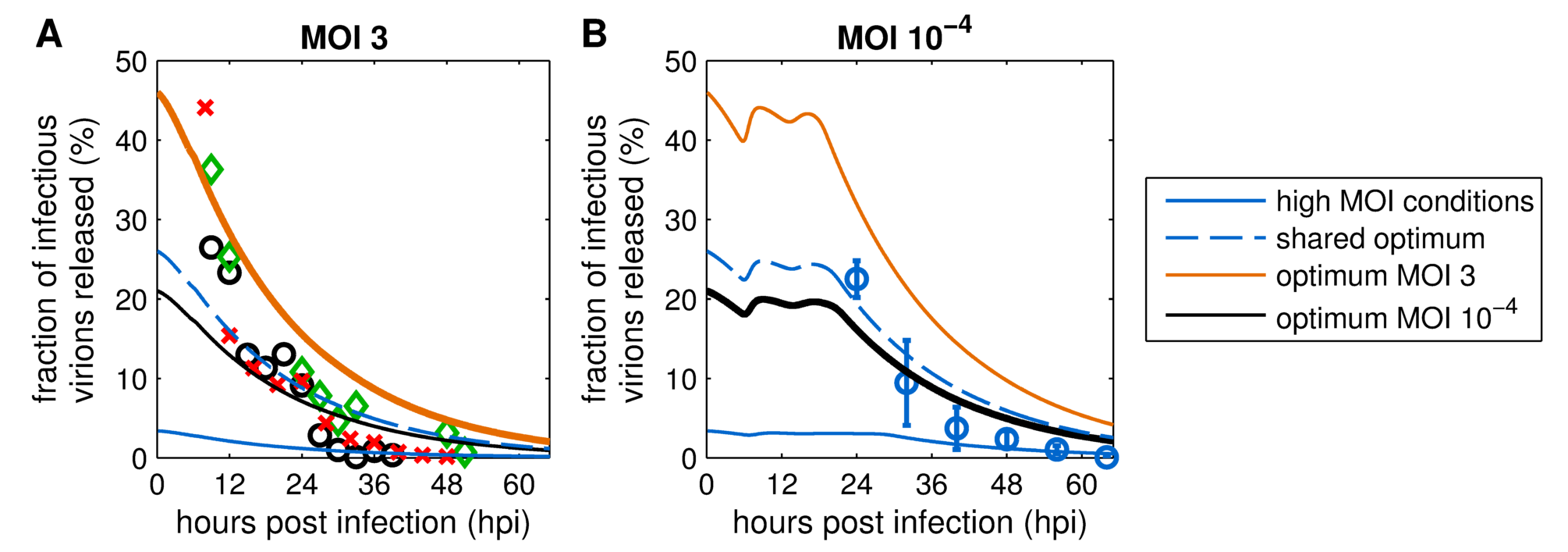

Supplement: S5 Fig — Simulation of the extended model with an MOI of (A) 3 and (B) 10−4 based on TCID50 using different initial FIVRs. Various initial FIVRs were tested for their ability to improve the model prediction for virus release dynamics in low MOI infections. Simulation results were evaluated based on their deviation to the experimental data and showed different optima at MOI 3 (FPar(0) = 0.46) and MOI 10−4 (FPar(0) = 0.21). The shared optimum (FPar(0) = 0.26) was determined by summing up deviations of both MOI 3 and 10−4 to obtain the initial FIVR resulting in the lowest error. (TIF) [file pcbi.1006819.s007.tif]
