# Supplementary material for: Multiscale modeling of influenza A virus replication in cell cultures predicts infection dynamics for highly different infection conditions
Source: PLoS Comput Biol. 2019 Feb 19;15(2):e1006819. doi: 10.1371/journal.pcbi.1006819 (PMC6396949; doi:10.1371/journal.pcbi.1006819)
Supplement: S1 Table — (DOCX) [file pcbi.1006819.s009.docx]

**S1 Table. Initial conditions for the extended multiscale model.**

| **MOI condition** | **Figure** | **Submodel** | **Non-zero initial conditions** |
| --- | --- | --- | --- |
| MOI 73 | Fig 2, 3, 4,  Fig 6 (dotted),  Fig S1, S3 | intracellular model | $V^{\text{Ex}}\left(0\right)=73\text{ virions}$ |
|  |  |  | **** |
|  |  | reduced intracellular model | $Vp^{\text{Cyt}}\left(0\right)=297.84\text{ molecules}$ |
|  |  |  | **** |
|  |  | extracellular model | $T\left(0\right)=4.24\times10^{5}\text{ cells}/\text{mL}$ $T_{\text{A}}\left(0\right)=2.63\times10^{4}\text{ cells}/\text{mL}$ $I\left(0\right)=1.75\times10^{5}\text{ cells}/\text{mL}$ $I_{\text{A}}\left(0\right)=3.53\times10^{3}\text{ cells}/\text{mL}$ $V\left(0\right)=3.1\times10^{7}\text{ virions}/\text{mL}$         |
|  |  |  |  |
|  |  |  |  |
|  |  |  |  |
|  |  |  |  |
|  |  |  |  |
| MOI 3 | Fig 5A-C (dashed),  Fig 6 (dash-dotted) | intracellular model | $V^{\text{Ex}}\left(0\right)=3\text{ virions}$ |
|  |  |  | **** |
|  |  | reduced intracellular model | $Vp^{\text{Cyt}}\left(0\right)=12.24\text{ molecules}$ |
|  |  |  | **** |
|  |  | extracellular model | $T\left(0\right)=5.99\times10^{5}\text{ cells}/\text{mL}$ $T_{\text{A}}\left(0\right)=0\text{ cells}/\text{mL}$ $I\left(0\right)=0\text{ cells}/\text{mL}$ $I_{\text{A}}\left(0\right)=0\text{ cells}/\text{mL}$ $V\left(0\right)=1.8\times10^{6}\text{ virions}/\text{mL}$         |
|  |  |  |  |
|  |  |  |  |
|  |  |  |  |
|  |  |  |  |
| MOI 10^-4^ | Fig 5D-F (dashed),  Fig 6 (solid) | intracellular model | $V^{\text{Ex}}\left(0\right)=1\text{ virion}$ |
|  |  |  | **** |
|  |  | reduced intracellular model | $Vp^{\text{Cyt}}\left(0\right)=8\text{ molecules}$ |
|  |  |  | **** |
|  |  | extracellular model | $T\left(0\right)=5.99\times10^{5}\text{ cells}/\text{mL}$ $T_{\text{A}}\left(0\right)=0\text{ cells}/\text{mL}$ $I\left(0\right)=0\text{ cells}/\text{mL}$ $I_{\text{A}}\left(0\right)=0\text{ cells}/\text{mL}$ $V\left(0\right)=59.9\text{ virions}/\text{mL}$         |
