# Supplementary material for: Multiscale modeling of influenza A virus replication in cell cultures predicts infection dynamics for highly different infection conditions
Source: PLoS Comput Biol. 2019 Feb 19;15(2):e1006819. doi: 10.1371/journal.pcbi.1006819 (PMC6396949; doi:10.1371/journal.pcbi.1006819)
Supplement: S2 Table — (DOCX) [file pcbi.1006819.s010.docx]

**S2 Table. Parameters of the intracellular model.**

| **Parameter** | **Description** | **Value** | **Unit** | **Source** |
| --- | --- | --- | --- | --- |
| **** | number of high-affinity binding sites | 150 | sites | [4] |
| **** | number of low-affinity binding sites | 1000 | sites | [4] |
| **** | distance between two adjacent ribosomes | 160 | nucleotides | [5] |
| **** | fraction of fusion-competent virions | 0.51 | − | [1] |
| **** | fraction of M2-encoding mRNAs | 0.02 | − | based on ratio  of M2 to M1 |
| **** | fraction of NEP-encoding mRNAs | 0.125 | − | [6] |
| **** | attachment to high-affinity binding sites | 8.09×10^-2^ | site^-1^·h^-1^ | adjusted to data in reference [4] |
| **** | attachment to low-affinity binding sites | 4.55×10^-4^ | site^-1^·h^-1^ | adjusted to data in reference [4] |
| **** | binding of M1 to nuclear vRNPs | 1.09×10^-6^ | moluecules^-1^·h^-1^ | model fit in Figure S1 |
| **** | binding of NP to  RdRp-RNA complexes | 3.01×10^-4^ | moluecules^-1^·h^-1^ | [1] |
| **** | binding of RdRp-complexes to RNA | 1 | moluecules^-1^·h^-1^ | [1] |
| **** | degradation of mRNA | 0.63 | h^-1^ | model fit in Figure S1 |
| **** | degradation of nascent cRNA/vRNA | 36.36 | h^-1^ | [1] |
| **** | degradation of RNPs | 0.09 | h^-1^ | [1] |
| **** | degradation of RdRp-RNA complexes | 4.25 | h^-1^ | [1] |
| **** | endocytosis | 4.8 | h^-1^ | [1] |
| **** | equilibrium constant of high-affinity sites | 1.13×10^-2^ | sites^-1^ | [4] |
| **** | equilibrium constant of low-affinity sites | 8.33×10^-5^ | sites^-1^ | [4] |
| **Parameter** | **Description** | **Value** | **Unit** | **Source** |
| **** | fusion with endosomes | 0.31 | h^-1^ | model fit in Figure S1 |
| **** | nuclear import | 6 | h^-1^ | [8] |
| **$K_{\text{R}}$** | mRNA synthesis inhibition constant | 1.1×10^7^ | molecules | model fit in Figure S1 |
| **** | formation of  RdRp-complexes | 1 | moluecules^-2^·h^-1^ | assuming rapid complex formation |
| **** | virion release/budding | 1270 | virions·h^-1^ | model fit in Figure S1 |
| **** | reduction of infectious release | 5.17×10^-2^ | h^-1^ | model fit in Figure S1 |
| **** | cRNA synthesis | 0.9 | h^-1^ | model fit in Figure S1 |
| **** | mRNA synthesis | 1.73×10^5^ | nucleotides·h^-1^ | model fit in Figure S1 |
| **** | protein synthesis | 64800 | nucleotides·h^-1^ | [9] |
| **** | vRNA synthesis | 8.33 | h^-1^ | model fit in Figure S1 |
| **** | influence of viral components on release | 1250 | virions | model fit in Figure S1 |
| **** | length of  segment 1’s mRNA | 2320 | nucleotides | [10] |
| **** | length of  segment 2’s mRNA | 2320 | nucleotides | [10] |
| **** | length of  segment 3’s mRNA | 2211 | nucleotides | [10] |
| **** | length of  segment 4’s mRNA | 1757 | nucleotides | [10] |
| **** | length of  segment 5’s mRNA | 1540 | nucleotides | [10] |
| **** | length of  segment 6’s mRNA | 1392 | nucleotides | [10] |
| **** | length of segment 7’s unspliced mRNA | 1005 | nucleotides | [10] |
| **** | length of segment 8’s unspliced mRNA | 868 | nucleotides | [10] |
| **** | average length  of a vRNA | 1700 | nucleotides | based on  reference [10] |
| **** | number of RdRp-complexes in a virion | 45 | molecules·  virion^-1^ | [10] |
| **Parameter** | **Description** | **Value** | **Unit** | **Source** |
| **** | number of HA molecules in a virion | 500 | molecules·  virion^-1^ | [10] |
| **** | number of NP molecules in a virion | 1000 | molecules·  virion^-1^ | [10] |
| **** | number of NA molecules in a virion | 100 | molecules·  virion^-1^ | [10] |
| **** | number of M1 molecules in a virion | 3000 | molecules·  virion^-1^ | [10] |
| **** | number of M2 molecules in a virion | 40 | molecules·  virion^-1^ | [10] |
| **** | number of NEP molecules in a virion | 165 | molecules·  virion^-1^ | [10] |
| **** | nucleotides bound by one M1 molecule | 200 | nucleotides | [11] |
| **** | nucleotides bound by one NEP molecule | 1700 | nucleotides | adjusted to data in reference [12] |
| **** | nucleotides bound by one NP molecule | 24 | nucleotides | [12] |

**Supplementary references**

1. Heldt FS, Frensing T, Reichl U. Modeling the intracellular dynamics of influenza virus replication to understand the control of viral RNA synthesis. Journal of Virology. 2012;86(15): 7806-7817.
2. Heldt FS, Frensing T, Pflugmacher A, Gröpler R, Peschel B, Reichl U. Multiscale modeling of influenza A virus infection supports the development of direct-acting antivirals. PLoS Computational Biology. 2013;9(11): e1003372.
3. Frensing T, Kupke SY, Bachmann M, Fritzsche S, Gallo-Ramirez LE, Reichl U. Influenza virus intracellular replication dynamics, release kinetics, and particle morphology during propagation in MDCK cells. Applied Microbiology and Biotechnology. 2016;100(16):7181-7192.
4. Nunes-Correia I, Ramalho-Santos J, Nir S, de Lima MCP. Interactions of influenza virus with cultured cells: Detailed kinetic modeling of binding and endocytosis. Biochemistry. 1999;38(3): 1095-1101.
5. Arava Y, Wang YL, Storey JD, Liu CL, Brown PO, Herschlag D. Genome-wide analysis of mRNA translation profiles in Saccharomyces cerevisiae. Proceedings of the National Academy of Sciences of the United States of America. 2003;100: 3889-3894.
6. Robb NC, Jackson D, Vreede FT, Fodor E. Splicing of influenza A virus NS1 mRNA is independent of the viral NS1 protein. Journal of General Virology. 2010;91: 2331-2340.
7. Amorim MJ, Bruce EA, Read EKC, Foeglein A, Mahen R, Stuart AD, et al. A Rab11-and Microtubule-Dependent Mechanism for Cytoplasmic Transport of Influenza A Virus Viral RNA. Journal of Virology. 2011;85: 4143-4156.
8. Babcock HP, Chen C, Zhuang XW. Using single-particle tracking to study nuclear trafficking of viral genes. Biophysical Journal. 2004;87: 2749-2758.
9. Spirin, AS. Ribosome structure and protein biosynthesis. The Benjamin/Cummings Publishing Company. 1986
10. Lamb RA, Krug RM. Orthomyxoviridae: the viruses and their replication. In: Knipe DM, Howley PM, Griffin EG, editors. Fields virology, 4th edition. Lippincott Williams & Wilkins; 2001. p.1487-1531
11. Wakefield L, Brownlee GG. Rna-Binding Properties of Influenza-a Virus Matrix Protein M1. Nucleic Acids Research. 1989;17: 8569-8580.
12. Portela A, Digard P. The influenza virus nucleoprotein: a multifunctional RNA-binding protein pivotal to virus replication. Journal of General Virology. 2002;83: 723-734.
13. Schulze-Horsel J, Schulze M, Agalaridis G, Genzel Y, Reichl U. Infection dynamics and virus-induced apoptosis in cell culture-based influenza vaccine production-Flow cytometry and mathematical modeling. Vaccine. 2009;27: 2712-2722.
14. Frensing T, Pflugmacher A, Bachmann M, Peschel B, Reichl U. Impact of defective interfering particles on virus replication and antiviral host response in cell culture-based influenza vaccine production. Applied Microbiology and Biotechnology. 2014;98:8999-9008.
