# Supplementary material for: Multiscale modeling of influenza A virus replication in cell cultures predicts infection dynamics for highly different infection conditions
Source: PLoS Comput Biol. 2019 Feb 19;15(2):e1006819. doi: 10.1371/journal.pcbi.1006819 (PMC6396949; doi:10.1371/journal.pcbi.1006819)
Supplement: S3 Table — (DOCX) [file pcbi.1006819.s011.docx]

**S3 Table. Parameters of the extracellular model.**

| **Parameter** | **Description** | **Value** | **Unit** | **Source** |
| --- | --- | --- | --- | --- |
| **** | maximum cell growth rate | 0.03 | h^-1^ | [13] |
| **** | number of high-affinity binding sites | 150 | sites·cell^-1^ | [4] |
| **** | number of low-affinity binding sites | 1000 | sites·cell^-1^ | [4] |
| **** | ratio of infected cells to fused virions | 1 | cells·virion^-1^ | [2] |
| **$K_{\text{I}}$** | maximum apoptosis rate of infected cell | 0.11 | h^-1^ | model fit in Figure S1 |
| **** | apoptosis rate of uninfected cells | 6.97×10^-3^ | h^-1^ | model fit in Figure S1 |
| **** | attachment to high-affinity binding sites | 3.32×10^-8^ | mL·sites^-1^·h^-1^ | adjusted to data  in reference [4] |
| **** | attachment to low-affinity binding sites | 1.85×10^-10^ | mL·sites^-1^·h^-1^ | adjusted to data  in reference [4] |
| **** | degradation/clearance of infectious virions | 1.15×10^-2^ | h^-1^ | model fit in Figure S1 |
| **** | endocytosis | 4.8 | h^-1^ | [1] |
| **** | equilibrium constant of high-affinity sites | 4.48×10^-9^ | mL·sites^-1^ | [4] |
| **** | equilibrium constant of low-affinity sites | 3.32×10^-11^ | mL·sites^-1^ | [4] |
| **** | fusion with endosomes | 0.31 | h^-1^ | model fit in Figure S1 |
| **** | lysis of apoptotic cells | 9.34×10^-3^ | h^-1^ | model fit in Figure S1 |
| **** | maximum cell concentration | 1×10^6^ | cells·mL^-1^ | maximum cell concentration observed in control flasks |
| **** | time after cell infection at which the rate of virus-induced apoptosis reaches its half-maximum | 19.8 | h | model fit in Figure S1 |
| **** | distribution factor of the virus-induced apoptosis rate | 0.76 | h^-1^ | model fit in Figure S1 |

**Supplementary references**

1. Heldt FS, Frensing T, Reichl U. Modeling the intracellular dynamics of influenza virus replication to understand the control of viral RNA synthesis. Journal of Virology. 2012;86(15): 7806-7817.
2. Heldt FS, Frensing T, Pflugmacher A, Gröpler R, Peschel B, Reichl U. Multiscale modeling of influenza A virus infection supports the development of direct-acting antivirals. PLoS Computational Biology. 2013;9(11): e1003372.
3. Frensing T, Kupke SY, Bachmann M, Fritzsche S, Gallo-Ramirez LE, Reichl U. Influenza virus intracellular replication dynamics, release kinetics, and particle morphology during propagation in MDCK cells. Applied Microbiology and Biotechnology. 2016;100(16):7181-7192.
4. Nunes-Correia I, Ramalho-Santos J, Nir S, de Lima MCP. Interactions of influenza virus with cultured cells: Detailed kinetic modeling of binding and endocytosis. Biochemistry. 1999;38(3): 1095-1101.
5. Arava Y, Wang YL, Storey JD, Liu CL, Brown PO, Herschlag D. Genome-wide analysis of mRNA translation profiles in Saccharomyces cerevisiae. Proceedings of the National Academy of Sciences of the United States of America. 2003;100: 3889-3894.
6. Robb NC, Jackson D, Vreede FT, Fodor E. Splicing of influenza A virus NS1 mRNA is independent of the viral NS1 protein. Journal of General Virology. 2010;91: 2331-2340.
7. Amorim MJ, Bruce EA, Read EKC, Foeglein A, Mahen R, Stuart AD, et al. A Rab11-and Microtubule-Dependent Mechanism for Cytoplasmic Transport of Influenza A Virus Viral RNA. Journal of Virology. 2011;85: 4143-4156.
8. Babcock HP, Chen C, Zhuang XW. Using single-particle tracking to study nuclear trafficking of viral genes. Biophysical Journal. 2004;87: 2749-2758.
9. Spirin, AS. Ribosome structure and protein biosynthesis. The Benjamin/Cummings Publishing Company. 1986
10. Lamb RA, Krug RM. Orthomyxoviridae: the viruses and their replication. In: Knipe DM, Howley PM, Griffin EG, editors. Fields virology, 4th edition. Lippincott Williams & Wilkins; 2001. p.1487-1531
11. Wakefield L, Brownlee GG. Rna-Binding Properties of Influenza-a Virus Matrix Protein M1. Nucleic Acids Research. 1989;17: 8569-8580.
12. Portela A, Digard P. The influenza virus nucleoprotein: a multifunctional RNA-binding protein pivotal to virus replication. Journal of General Virology. 2002;83: 723-734.
13. Schulze-Horsel J, Schulze M, Agalaridis G, Genzel Y, Reichl U. Infection dynamics and virus-induced apoptosis in cell culture-based influenza vaccine production-Flow cytometry and mathematical modeling. Vaccine. 2009;27: 2712-2722.
14. Frensing T, Pflugmacher A, Bachmann M, Peschel B, Reichl U. Impact of defective interfering particles on virus replication and antiviral host response in cell culture-based influenza vaccine production. Applied Microbiology and Biotechnology. 2014;98:8999-9008.
